# Traditional Chinese medicine for colorectal cancer treatment: potential targets and mechanisms of action

**DOI:** 10.1186/s13020-023-00719-7

**Published:** 2023-02-13

**Authors:** Jin-Fang Chen, Shi-Wei Wu, Zi-Man Shi, Bing Hu

**Affiliations:** 1 Institute of Traditional Chinese Medicine in Oncology, Longhua Hospital, Shanghai University of Traditional Chinese Medicine, 200032 Shanghai, People’s Republic of China; 2 Department of Oncology, Longhua Hospital, Shanghai University of Traditional Chinese Medicine, 200032 Shanghai, People’s Republic of China

**Keywords:** Colorectal cancer, Traditional Chinese medicine, Signaling pathway, Mechanism of action

## Abstract

Colorectal cancer (CRC) is a disease with complex pathogenesis, it is prone to metastasis, and its development involves abnormalities in multiple signaling pathways. Surgery, chemotherapy, radiotherapy, target therapy, and immunotherapy remain the main treatments for CRC, but improvement in the overall survival rate and quality of life is urgently needed. Traditional Chinese medicine (TCM) has a long history of preventing and treating CRC. It could affect CRC cell proliferation, apoptosis, cell cycle, migration, invasion, autophagy, epithelial–mesenchymal transition, angiogenesis, and chemoresistance by regulating multiple signaling pathways, such as PI3K/Akt, NF-κB, MAPK, Wnt/β-catenin, epidermal growth factor receptors, p53, TGF-β, mTOR, Hedgehog, and immunomodulatory signaling pathways. In this paper, the main signaling pathways and potential targets of TCM and its active ingredients in the treatment of CRC were systematically summarized, providing a theoretical basis for treating CRC with TCM and new ideas for further exploring the pathogenesis of CRC and developing new anti-CRC drugs.

## Introduction

Colorectal cancer (CRC) is the third most common malignancy worldwide, with morbidity and mortality rates of 10% and 9.4%, respectively [1]. In the past decade, the incidence of CRC in people under 50 years of age has been increasing year by year [2]. The incidence rate of CRC is estimated to increase by about 80% worldwide by 2035 [3]. Besides, it is the second most common tumor diagnosed in women and the third in men. It is worth mentioning that the incidence and mortality rate of CRC in women are approximately 25% lower than those in men [4]. CRC has become one of the risk factors threatening public health [5]. The clinical symptoms of CRC are intestinal dysfunction, mainly including abdominal pain, abdominal distension, increased frequency of bowel movements, bowel discomfort, and rectal bleeding [6]. Since the early stage of clinical symptoms are not obvious, most patients are often in the advanced stage after diagnosis. Patients with advanced CRC may develop intestinal obstruction and other systemic symptoms (such as weight loss and anemia), and even metastasis to lymph nodes, liver, lung, bone and other sites, which eventually leads to death [7]. Therefore, the study for effective treatment has become a research hotspot.

The pathogenesis of CRC is complex and diverse, and it is influenced by the interaction and influence of risk factors, such as genetics, diet, lifestyle, and inflammatory bowel disease (IBD) [8–10]. A long-term diet rich in red meat and lacking in fruits and vegetables may cause an increased incidence of CRC [11]. Most CRCs arise from cancer stem cells (CSC) within the colonic epithelium, accumulating progressive genetic and epigenetic alterations. These alterations result in impaired gene expression or function, thus favoring the activation of oncogenes and the downregulation of tumor suppressor genes [12]. The pathological features of CRC involve regional lymph node and distant metastasis, accompanied by molecular markers, such as BRAF, Kirsten rat sarcoma (K-Ras), microsatellite-unstable/instability (MSI) and caudal-related homeobox 2 (CDX2) [13]. BRAF, as a proto-oncogene, is involved in encoding serine/threonine protein kinases of the mitogen-activated protein kinase (MAPK) pathway; it acts as a direct effector of RAS; and it is involved in promoting tumor growth and survival [14]. Many studies have shown that BRAF mutations (BRAF-mt) are associated with prognosis and metastasis in CRC and may be influenced by MSI status [15–18]. K-Ras protein, encoded by the proto-oncogene k-RAS, is an important component of the MAPK pathway [19]. K-RAS mutation (KRAS-mt) confers tumor cell growth at lower glucose concentrations than those required by normal cells and strongly promotes tumor cell growth [20]. CDX2 encodes transcription factors involved in regulating intestinal differentiation and development [21]. It also acts as a tumor suppressor, and it is associated with the pathogenesis of distal colon tumors [22, 23].

Currently, surgery, radiotherapy, chemotherapy, immunotherapy, and targeted therapy are the main treatments for CRC, but problems, such as surgical sequelae, chemotherapy resistance, toxic side effects, high metastasis, and recurrence rates, seriously affect the quality of life of patients [24, 25]. Traditional Chinese medicine (TCM), as a predominant source of natural medicines and herbal products, are essential sources for exploiting anti-CRC drugs [26]. As one of the effective means to treat CRC, TCM could exert anti-CRC effects in multiple targets and pathways while ameliorating the toxic side effects elicited by surgery chemotherapy, radiotherapy, target therapy, and immunotherapy and prolonging patients’ survival time [27, 28]. Experimental studies have shown that TCM and its components could effectively inhibit CRC cell proliferation, induce apoptosis, block cell cycle, promote cell autophagy, and inhibit angiogenesis; it also plays an anti-CRC role in cooperation radiotherapy and chemotherapy [29–32]. Regulating signaling pathways is one of the important mechanisms for CRC treatment. Exploring the mechanism of CRC-related signaling pathways could help further clarify the anti-CRC targets of TCM. Therefore, in this study, the research progress of the regulation of CRC-related signaling pathways by TCM and its active ingredients was systematically summarized, providing a reference for further studies on the prevention and treatment of CRC by TCM.

### Phosphatidylinositol 3-kinase/protein kinase-B (PI3K/Akt) signaling pathway

**Fig. 1:**
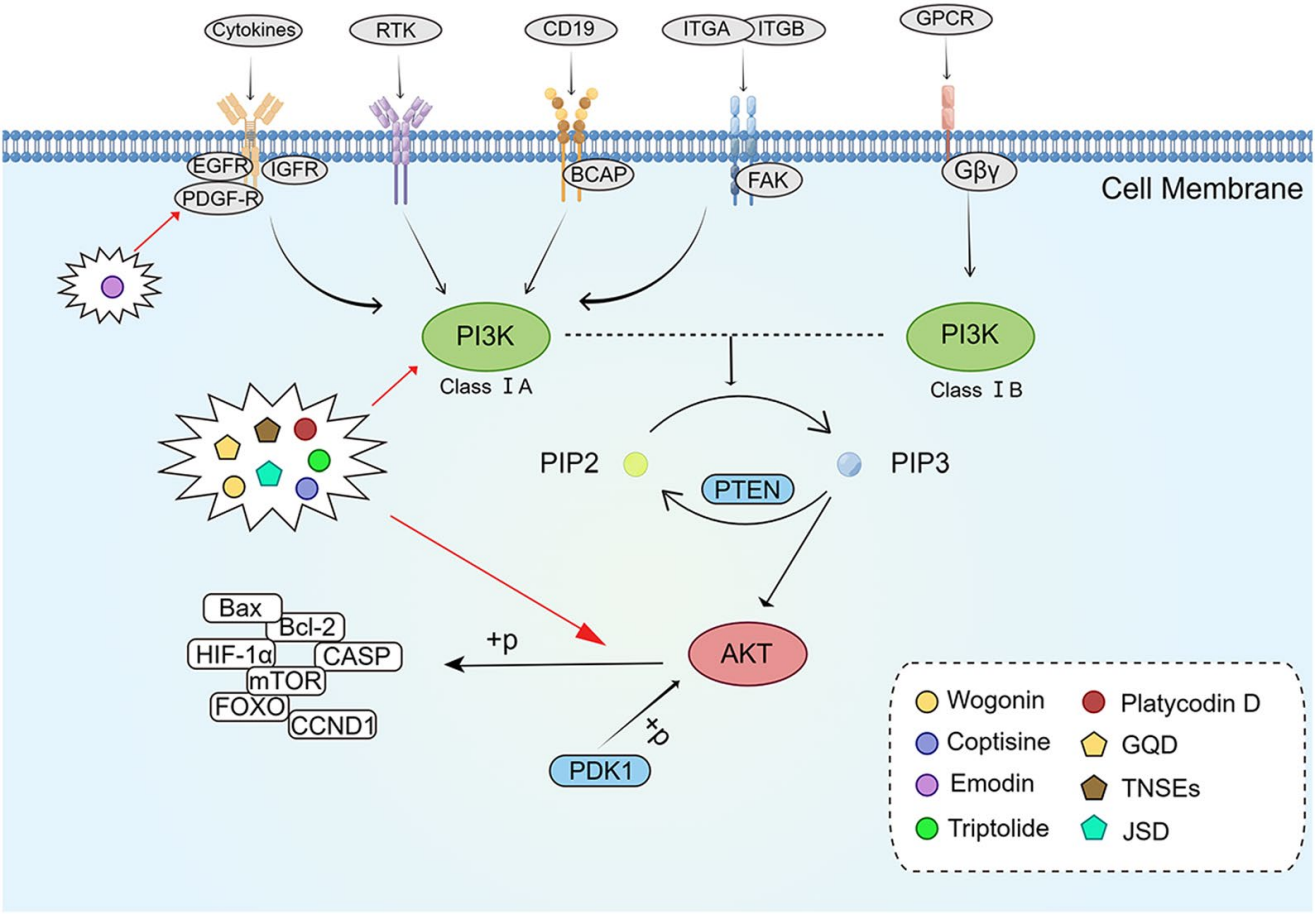
The active compounds of TCM and the Chinese herb formula act on the PI3K/Akt signaling pathway. *GOD* gegen qinlian decoction, *TNSEs* tounong powder, *JSD*, jiedu sangen decoction. The figure was created by Figdraw (https://www.figdraw.com/static/index.html#/)

The PI3K/Akt signaling pathway is one of the important intracellular signaling pathways and a major effector downstream of receptor tyrosine kinases (RTKs) and G protein coupled receptors [33, 34]. This pathway is stimulated by various oncogenes and growth factor receptors, including platelet-derived growth factor receptors, insulin like growth factor receptors, and epidermal growth factor receptors (EGFRs) [35]. The main proteins of this signaling pathway are PI3K and Akt [36]. Activation of PI3K promotes signal transduction cascades for tumor cell growth, survival, and metabolism [37]. Akt, as a serine-threonine kinase, is a major downstream target of PI3K, and it could directly respond to PI3K activation [38]. Akt/PKB kinase has three highly homologous isoforms: Akt1/PKBa, Akt2/PKBb, and Akt3/PKBg. Akt1 is involved in the regulation of tumor growth, tumor cell invasion, and chemoresistance, and it is the main isoform found in various cancers. Alterations in Akt2 could be observed in breast cancer, ovarian cancer, pancreatic cancer, and CRC, and it is associated with tumor cell invasion, metastasis, and survival. Akt3 is mainly expressed in melanoma, glioma, and some breast cancers, affecting tumor growth and drug resistance [39]. Several studies have demonstrated that the PI3K/Akt signaling pathway is aberrantly activated in many cancers, and that it is closely related to tumor cell proliferation, apoptosis, invasion, epithelial–mesenchymal transition (EMT), stem cell-like phenotype, and tumor drug resistance [40], in addition to being involved in tumor angiogenesis [41]. Therefore, targeting the PI3K/Akt signaling pathway could contribute to anti-CRC therapy (Fig. 1).

Wogonin (WOG) is a flavonoid compound found in *Scutellaria baicalensis* Georgi (Huang Qin), which has been proven to inhibit tumor cell growth and induce apoptosis [42, 43]. The expression of light chain 3-II (LC3II); Beclin-1; caspase-3, -8, and -9; and Bcl-2-associated X (Bax) protein was upregulated, whereas that of B-cell lymphoma-2 (Bcl-2) was downregulated in WOG-treated SW48 cells. Cell cycle was also arrested in the G2/M phase. In addition, the expression of p-PI3K, p-Akt, phosphorylated signal transducer and activator of transcription 3 (p-STAT3) in SW48 cells showed a concentration-dependent decrease while the expression of total PI3K, Akt and STAT3 was not significantly affected. The above results suggested that WOG may induce apoptosis and arrest cell cycle in SW48 cells through PI3K/Akt pathway[44]. Coptisine (COP), the main active ingredient in *Rhizoma coptidis* (Huang Lian), has antitumor activity [45, 46]. Huang et al. [47] found that COP initiated extrinsic apoptotic pathways in vitro by inhibiting the PI3K/Akt signaling pathway and thus upregulation of cleaved caspase-8 and  -3. Meanwhile, the expression levels of Cyclin D1 and Cyclin E were down-regulated, thus inducing G0/G1 phase cell cycle arrest. In HCT-116 CRC xenograft mice model, COP was found to inhibit tumor growth and effectively reverse the elevated serum markers carcinoembryonic antigen, carbohydrate antigen 19-9, and cytokeratin fragment antigen 21-1 in BALB/c nude mice. Emodin (EMD) is the main component of *Rheum palmatum* (Da Huang), which has been widely used in the treatment of various diseases [48]. Dai et al. [49] found that EMD inhibited the expression of VEGFR2, PI3K and p-Akt in HCT-116 cells and tumor-bearing mice, suggesting that EMD may inhibit human CRC cell growth, adhesion and migration by suppressing VEGFR2/PI3K/Akt signaling pathway. In addition, Liu et al. [50] showed that triptolide (TP), an extract of *Tripterygium wilfordii* Hook F. (Lei Gong Teng), could reduce the phosphorylation of p-Akt (Thr308) in HT29 cells and p-Akt (Ser473) in SW480 cells and exert anti-proliferative effects through the Akt signaling pathway Platycodin D (PD) is a triterpenoid saponin-like ingredient from the Chinese herb *Platycodon grandiflorum* (Jie Geng) with multiple biological effects [51–53]. Liu et al. [54] found that the combination of PD and cetuximab downregulated the phosphorylation of PI3K and Akt in HCT116 and LoVo cells both in vivo and in vitro, and increased the cytotoxic effect of cetuximab. The synergistic effect between PD and cetuximab was attenuated after application of Akt activator SC-79. The results mentioned above implied that PD may cause CRC cells to become more sensitive to cetuximab by inhibiting the PI3K/Akt signaling pathway. (Fig. 1).

Li et al. [55] found that Gegen Qinlian Decoction (GQD) could inhibit CT-26 CRC growth accompanied by upregulation of p-PI3K, p-Akt, phosphorylated forkhead box transcription factor O1 (p-FOXO1), and ankyrin repeat and BTB/POZ domain containing protein 1 (ABTB1). Fang et al. [56] found that Tounong powder extracts (TNSEs) induced LoVo cell growth inhibition in a dose- and time-dependent manner; significantly downregulated the expression of PI3K, p-AKT, phosphorylated mechanistic target of rapamycin (p-mTOR), and p-p70s6k1; and upregulated the expression of cleaved caspase-9 and -3. These findings suggested that TNSEs may inhibit LoVo cells through the PI3K/Akt signaling pathway. Sun et al. [57] found that the expression of hypoxia-inducible factor-1α (HIF-1α), PI3K, Akt/p-Akt, hexokinase II, and glucose transporter type 1 (GLUT1) was downregulated and that of caspase-3 and -9 was upregulated in 5-FU-resistant human CRC cells (HCT-8/5-FU) after the intervention of Jiedu Sangen decoction (JSD). HIF-1α silencing showed a significant decrease in the expression levels of PI3K, Akt, and p-Akt, accompanied by an upregulation of caspase-6 and -7 expression. This finding suggested that JSD inhibits glycolysis through the PI3K/Akt/HIF-1α signaling pathway to reverse 5-FU resistance and induce apoptosis to enhance antitumor activity (Fig. 1).

### Nuclear factor kappa-beta (NF-κB) signaling pathway

**Fig. 2:**
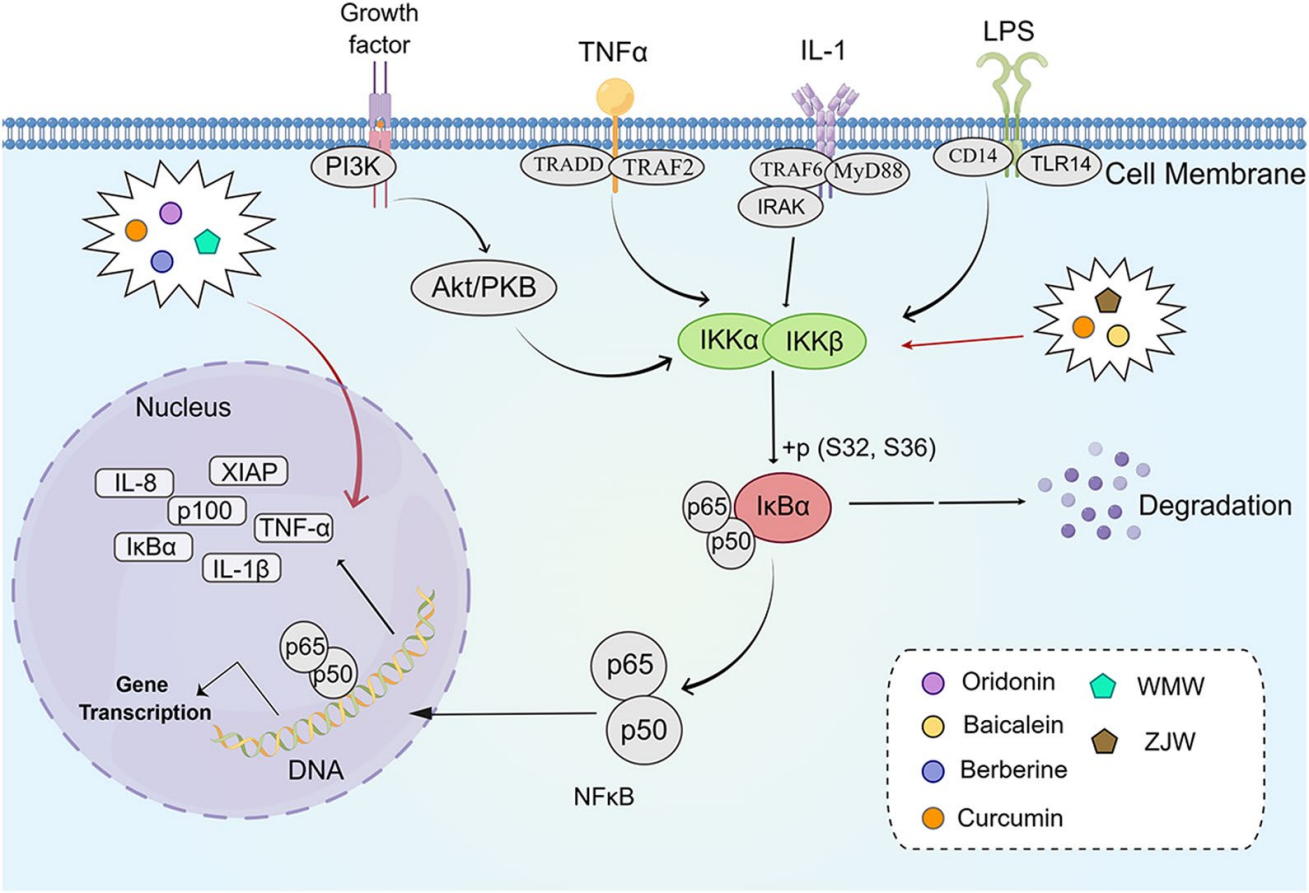
The active compounds of TCM and the Chinese herb formula act on the NF/κB signaling pathway. *WMW* Wu Mei Wan, *ZJW* Zuo Jin Wan. The figure was created by Figdraw (https://www.figdraw.com/static/index.html#/)

NF-κB is a ubiquitous transcription factor that could directly participate in mediating cyclotomic/neutral signaling and regulate the expression of various cytokines and cell adhesion molecules involved in inflammation and immune responses [58, 59]. The activation of NF-κB correlates with apoptosis, cell cycle, proliferation, differentiation, migration, and resistance to radiation/chemotherapy in tumor cells [60]. The five currently known members of the NF-κB family are as follows: p50/p105 (NF-κB1), p52/p100 (NF-κB2), c-Rel, RelB, and p65 (RelA); each protein has its Rel homologous structural domain that controls DNA binding, dimerization, and interaction with the repressor IκB [61]. In most quiescent cells, the IκB in the cytoplasm binds to NF-κB and inactivates it by overriding the nuclear localization sequence to block DNA binding and nuclear uptake [58]. The IκB kinase (IKK) complex contains a regulatory subunit IKKγ (NEMO), catalytic subunits IKKα and IKKβ, which are upregulated by the interaction of cell surface receptors, such as Toll-like receptor, T/B cell receptor, and tumor necrosis factor receptor with specific ligands [62]. IKK could cause IκB phosphorylation via ubiquitin-proteasome pathway degradation to activate NF-κB and cause nuclear translocation [63]. In the nucleus, NF-κB binds to the enhancer elements of the immunoglobulin kappa light chain (κB sites) in activated B cells, triggering the expression of downstream genes that lead to the progression of inflammation or cancer [64, 65]. In human CRC adenocarcinoma, NF-κB expression is proportional to the abnormal activity of K-Ras [66]. The activation of NF-κB was found to be decreased in SW620 cells after K-Ras knockdown, possibly through the Ras/extracellular signal-regulated kinase (ERK)/IκBα signaling pathway [67] (Fig. 2).

Oridonin (ORI) is a bioactive ingredient extracted from *Rabdosia rubescens* (Dong Ling Cao) [68]. It has been shown to have a therapeutic effect on various malignancies, including liver cancer, skin carcinoma, and osteoma [69, 70]. Jin et al. [71] found that the expression of HECT and RCC1-containing protein 5 was upregulated in LoVo and SW480 cells, whereas activating protein-1 (AP-1), NF-κB, and p38 were downregulated after ORI treatment. In-vivo studies confirmed that ORI first inhibited the expression of AP-1 and then downregulated the expression of p38 and NF-κB, suggesting that ORI may exert anti-CRC effects through the NF-κB signaling pathway and P38-dependent MAPK signaling pathway. Baicalein (BE) is one of the four major flavonoids found in *Scutellaria baicalensis* Georgi (Huang Qin), which has anti-inflammatory and anti-cancer effects [72]. Kim et al. [73] found that BE could inhibit the NF-κB signaling pathway and regulate apoptosis, migration, invasion, and inflammatory response in CRC cells through activation of peroxisome proliferator-activated receptor γ. Berberine (BBR) is a compound widely found in Chinese herbs, such as *Coptidis Rhizoma* (Huang Lian), *Phellodendron chinense* Schneid. (Huang Bai), and *Berberis silva-taroucana* Schneid. (Xiao Bo). Chen et al. [74] found that BBR reversed the upregulated protein expression of Ki-67 and β-catenin; downregulated the expression of interleukin-1β (IL-1β), tumor necrosis factor α (TNF-α), NF-κB, matrix metallopeptidase 9 (MMP9), Ereg, and Muc16 in AMO/DSS model mice; and regulated cell proliferation, angiogenesis, and invasion through the NF-κB signaling pathway by exerting anti-CRC effects. Curcumin, the major component of *Curcuma longa* L. (Jiang Huang), can inhibit the proliferation of CRC cell lines and enhance capecitabine-induced apoptosis in vitro. It also inhibited the expression of NF-κB and its regulated gene products cyclo-oxygenase-2 (COX2), Cyclin D1, c-Myc, MMP-9, intercellular adhesion molecule-1 (ICAM-1), C-X-C motif chemokine receptor 4 (CXCR4), Survivin, Bcl-2 and vascular endothelial growth factor (VEGF). In vivo studies showed that curcumin was able to inhibit the growth and distal metastasis of HCT-116 CRC, and this inhibition was enhanced when combined with capecitabine. According to the aforementioned findings, curcumin may increase capecitabine’s anti-proliferative, invasive, metastatic, angiogenic, and pro-apoptotic actions on CRC via suppressing the NF-κB signaling pathway [75]. (Fig. 2).

In addition, herbal compounds may exert anti-CRC effects through the NF-κB pathway. Wu Mei Wan (WMW) is a herbal compound commonly used in clinical practice for the treatment of febrile diseases and other gastrointestinal related diseases [76]. Jiang et al. [77] found that AOM/DSS-induced colitis-associated CRC (CAC) mice showed downregulation of p65 and p-STAT3 expression in colonic tissues and downregulation of interleukin 6 (IL-6), p65, and p-STAT3 expression in serum after WMW intervention. This finding suggested that WMW inhibited tumor cell proliferation and improved CAC symptoms in model mice through downregulation of the NF-κB/IL-6/STAT pathway. Zuo Jin Wan (ZJW) consists of *Coptidis Rhizoma* (Huang Lian) and *Evodia rutaecarpa* (Juss.) Benth. (Wu Zhu Wu) in a 6:1 ratio. Sui et al. [78] found that ZJW could downregulate the phosphorylation of Akt (Ser473) and NF-κB expression in HCT-116/L-OHP cells. Moreover, the above downregulation was attenuated after treatment with PI3K/Akt activator, suggesting that ZJW reverses drug resistance in human CRC cells by blocking the PI3K/Akt/NF-κB signaling pathway and enhances the anti-apoptotic effect of oxaliplatin (Fig. 2).

### Mitogen-activated protein kinase (MAPK) signaling pathway

**Fig. 3:**
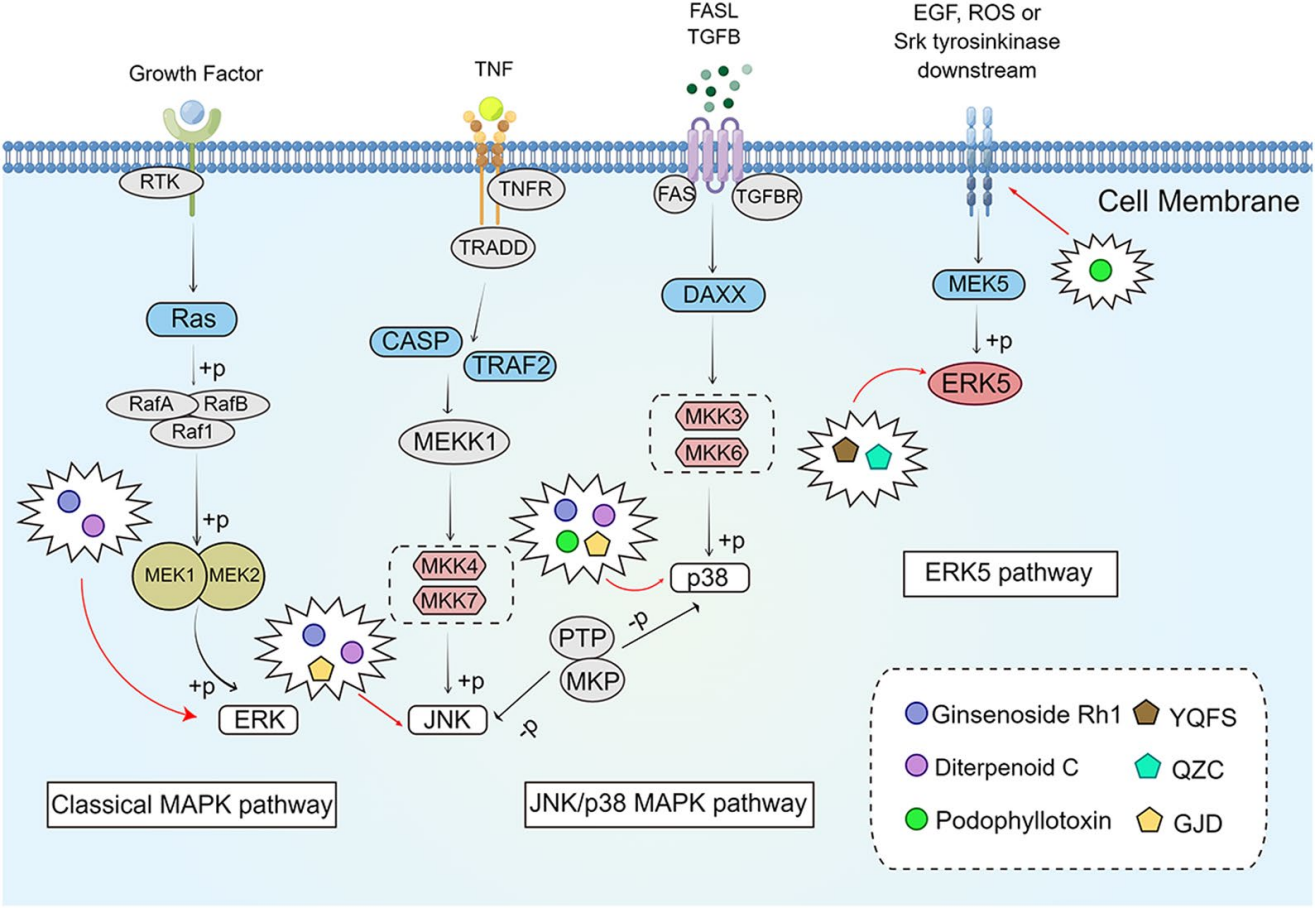
The active compounds of TCM and the Chinese herb formula act on the MAPK signaling pathway. *YQFS* Yi-Qi-Fu-Sheng, *QZC* Qizhen capsule, *GJD* Geijigajakyak decoction. The figure was created by Figdraw (https://www.figdraw.com/static/index.html#/)

MAPKs are serine-threonine kinases that could link extracellular signals to regulate cellular activities, such as cell proliferation, differentiation, migration, and apoptosis [79, 80]. The mammalian MAPK family includes ERK1/2; ERK3/4; ERK5; ERK7/8; Jun N-terminal kinase (JNK)1/2/3; and p38-α, -β, -γ, and -δ [81]. Each signal transduction axis of MAPK contains three components: MAPK, MAPK kinase (MAP2K), and MAPK kinase-kinase (MAP3K), which are named in accordance with their proximity to the nucleus [82]. Activation of MAPK could regulate transcription factors, such as ETS like-1 protein, c-Jun, transcription factor 2 (ATF2), and p53 [83]. The activation of MAPK pathway is the result of interactions between the kinase components [84]. In the MAPK/ERK pathway, phosphorylation of Raf (Raf-1, B-Raf, and A-Raf) activates MEK1/2, which leads to phosphorylation of ERK1/2 [79]. ERK1/2 shuttles from the cytoplasm to the nucleus and regulates gene expression by phosphorylating many transcription factors; the microtubule-associated proteins (MAP1, MAP2, and MAP4) in the cytoplasm are also targets of ERK1/2 kinases [85]. MEK and ERK1/2 are involved in cell survival, proliferation, and differentiation depending on their phosphorylated targets [86]. Activation of ERK/MAPK signaling pathway could promote tumor cell invasion and metastasis through upregulation of MMP expression [87]. JNK and p38 signaling pathways are activated by pro-inflammatory cytokines, such as TNF-α and IL-1β, or are involved in response to cellular stress [88]. MKK4 and MKK7, as representatives of MAP2K kinases, are JNK sub-pathways that are activated when MAP2K is triggered. Phosphorylation of these components activates JNK, which, in turn, phosphorylates AP-1 transcription factors, such as c-Jun, Fos, and Fos-related antigen1/2 (FRA1/2) [85]. AP-1 is associated with cell proliferation, survival, differentiation, inflammation, migration, and metastatic activities. Other downstream targets of JNK include the mitochondrial apoptosis regulator Bcl-2 family (Bcl-2, Bcl-xl, Bad, Bim, and Bax) and tumor suppressor p53, which are involved in the pro-apoptotic function of JNK [89]. In addition, JNK could promote cancer invasion and metastasis by promoting the expression of MMP7 and MMP9 [90]. The MAPK signaling pathway is an important regulator of tumor cell response to internal and external environmental stimuli. Therefore, modulation of the MAPK signaling pathway could help treat CRC (Fig. 3).

Ginsenoside Rh1 (Rh1), a compound of *Panax ginseng* C.A. Mey (Ren Shen)., has significant antitumor activity against human hepatocellular carcinoma, THP-1 acute monocytic leukemia cells, and astroglioma [91–93]. Lyu et al. [94] found that the expression of MMP1 and MMP3 was downregulated; the expression of tissue inhibitor of metalloproteinase 3 was upregulated; and the ratios of p-P38/P38, p-ERK1/2/ERK1-2, and p-JNK/JNK were decreased in SW620 cells after Rh1 intervention. They also showed the same trend in xenograft tumor-bearing mice, suggesting that Rh1 could exert anti-CRC effects through the MAPK signaling pathway. Diterpenoid C is an ingredient of *Curcuma longa *L. (Jiang Huang) [95]. Shen et al. [96] found that diterpenoid C inhibited the phosphorylation of ERK, JNK, and p38 MAPK and promoted the cleavage of caspase-3 in SW620 cells, suggesting that diterpenoid C exerts antiproliferative and pro-apoptotic effects through the MAPK signaling pathway. Podophyllotoxin (PT) is an active ingredient extracted from *Podophyllum peltatum* (Gui Jiu), which is highly cytotoxic to various cancer cells [97–99]. Lee et al. [100] found that PT intervention increased the level of reactive oxygen species (ROS), upregulated the expression of ER stress markers GRP78 and CHOP, and increased the phosphorylation of p38 MAPK in HCT-116 cells, suggesting that PT could induce the p38 MAPK signaling pathway and ER stress-mediated apoptosis through upregulation of ROS in HCT-116 cells, accompanied by G2/M phase cell-cycle arrest (Fig. 3).

In addition, Deng et al. [101] found that the Chinese herbal formula Yi-Qi-Fu-Sheng (YQFS) exerted anti-CRC effects in vivo and in vitro. In vivo, YQFS significantly inhibited tumor growth by downregulating the expression of MMP2 and MMP9 through inhibiting the ERK pathway. In vitro, YQFS inhibited HCT-116 cell invasion and migration and induced apoptosis by targeting ERK phosphorylation to regulate the ERK/MAPK pathway and its downstream factors. Guo et al. [102] found that Qizhen capsule (QZC), a commonly used clinical anticancer agent, could upregulate the levels of cleaved caspase-9 and -3, Bax, and nonsteroidal anti-inflammatory drug-activated gene-1/growth differentiation factor-15 (NAG-1/GDF15) and the phosphorylated expression of mTOR, MAPK/ERK, AMP-activated protein kinase (AMPK), and p38 and downregulate the expression of Bcl-2. QZC could exert a pro-apoptotic effect on HCT-116 cells by activating the MAPK/ERK signaling pathway mediating the upregulation of NAG-1/GDF15. Lee et al. [103] found that the expression of p-JNK and p-p38 MAPK was downregulated in HCT-116 cells after Geijigajakyak decoction (GJD) intervention, suggesting that GJD plays a role in anti-CRC invasion through JNK and p38 MAPK signaling pathways (Fig. 3).

### Wnt/β-catenin signaling pathway

**Fig. 4:**
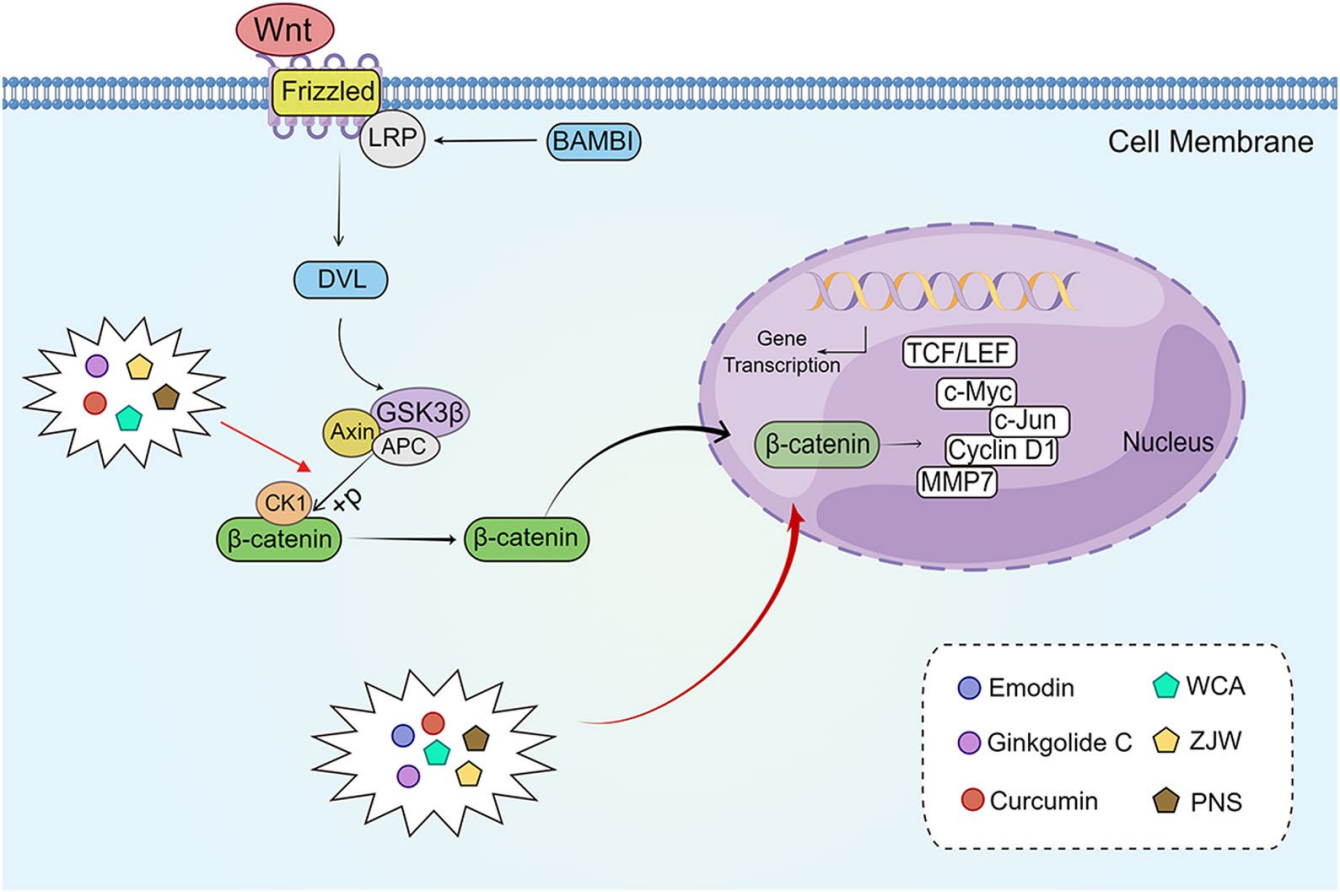
The active compounds of TCM and the Chinese herb formula act on the Wnt/β-catenin signaling pathway. *WCA* Weichang’an, *ZJW* Zuo Jin Wan, *PNS* Pai-Nong-San. The figure was created by Figdraw (https://www.figdraw.com/static/index.html#/)

The Wnt signaling pathway could be divided into canonical (β-catenin-dependent activity) and non-canonical (β-catenin-independent activity) Wnt pathways [104]. The β-catenin-dependent signaling pathway is mainly involved in the regulation of cell proliferation, and the β-catenin-independent signaling pathway is associated with cell mobility and polarity [105, 106]. The canonical Wnt/β-catenin pathways involve several interacting proteins, including the serine/threonine kinases glycogen synthase kinase 3beta (GSK3β) and casein kinase 1 (CK1), the tumor suppressors Axin and adenomatous polyposis coli (APC), and the E3 ubiquitin ligase β-TrCP, together forming the destruction complex; Axin is the backbone of the complex, interacting with β-catenin, GSK3β, and CK1 [107]. Activation of canonical Wnt/β-catenin pathways allows β-catenin to accumulate in the cytoplasm and further translocate to the nucleus as a transcriptional co-activator of T-cell transcription factor (TCF)/lymphoid enhancer factor (LEF) [108, 109]. The activity of Wnt/β-catenin signaling is related to the level of β-catenin in the nucleus, and regulation of the level of β-catenin is the basis of controlling the Wnt/β-catenin signaling pathway [110]. β-catenin binds to its transcription factors and causes transcription of target genes, such as c-Myc, cyclin D1, and MMPs [111]. Aberrant activation of the Wnt/β-catenin signaling pathway contributes to cell proliferation, differentiation, and renewal of tumor stem cells [112]. In most CRCs, overexpression of target genes in the Wnt/β-catenin signaling pathway induces dysregulation of the CRC cell cycle, along with accelerated invasion and metastasis [113, 114]. Regulation of the Wnt/β-catenin signaling pathway contributes to anti-CRC proliferation and metastasis effects (Fig. 4).

Several studies have confirmed that emodin induces apoptosis and inhibits the migration and invasion of colon cancer cells [115, 116]. Pooja et al. [117] showed that emodin significantly inhibited the mRNA expression of CTNNB1 (β-catenin) and transcription factor-7-like-2 (TCF7L2) and the expression of cyclin D1, c-Myc, snail, vimentin, MMP2, and MMP9, the downstream targets of Wnt signaling in human colon cancer cells. Further investigation of the inhibitory mechanism of emodin on Wnt signaling revealed that emodin downregulated coactivator p300 and upregulated transcriptional repressor HBP1 at the mRNA and protein levels, suggesting that emodin exerts anti-CRC effects by inhibiting the Wnt signaling pathway. Ginkgolide C (GGC) is a diterpenoid lactone compound isolated from *Ginkgo biloba* L. (Yin Xing Ye) [118, 119]. Yang et al. [120] found that GGC downregulated the Wnt/β-catenin signaling cascade and the expression of MMP2, MMP9, Wnt3a, β-catenin, and β-catenin downstream signals (Axin-1, p-GSK3β, and β-TrCP) and their target genes (c-myc, cyclin D1, and survivin) in HT-29 cells, suggesting that GGC may play a role in the anti-proliferation, anti-invasion, anti-migration and pro-apoptosis of CRC cells by targeting the Wnt/β-catenin signaling pathway. Curcumin, the main component of *Curcuma longa* L. (Jiang Huang), has also shown antitumor activity [121–123]. Jiang et al. [124] found that curcumin could significantly upregulate the expression of CDX2; downregulate the expression of Wnt3a and Wnt downstream signaling genes, such as c-Myc, surviving, and cyclin D1; and decrease the nuclear translocation of β-catenin in SW620 cells. After silencing the CDX2, the inhibitory effects of curcumin on c-Myc, surviving, and cyclin D1 were significantly reduced. These results suggested that curcumin inhibits the Wnt/β-catenin signaling pathway by activating *CDX2* and then exerts anti-proliferative and pro-apoptotic effects on SW620 cells. In addition, baicalein, the active ingredient in *Scutellaria baicalensis* Georgi, alleviates oxaliplatin-induced peripheral neuropathy, possibly also through the Wnt/β-catenin signaling pathway [125] (Fig. 4).

Tao et al. [126] found that the Chinese herbal formula Weichang’an (WCA) dose-dependently upregulated rho GTPase activating protein 25 (ARHGAP25) expression in HCT-116 cells while downregulating the expression of MMP7, MMP9, zinc finger E-box binding homeobox 1 (ZEB1), and β-catenin, suggesting that inhibition of the Wnt/β-catenin signaling pathway is the mechanism by which WCA exerts its anti-CRC migratory and invasive effects in vitro. Pan et al. [127] found that the expression of 5-hydroxytryptamine receptor D (5-HTR1D) and axin-1 was dose-dependently increased, whereas that of dishevelled 2 (Dvl2), p-GSK-3β, lymphoid enhancer-binding factor 1 (LEF1), transcription factor 4 (TCF4), MMP2, MMP7, ICAM-1, and CXCR4 was dose-dependently decreased in SW408 cells treated with Zuo Jin Wan (ZJW) extract. This finding indicated that the anti-CRC activity of ZJW extract could be achieved by inhibiting the 5-HTR1D-Wnt/β-catenin signaling pathway. In addition, Zhang et al. [128] found that Pai-Nong-San (PNS) was protective against AOM/DSS-induced colonic injury and able to downregulate p-GSK3β, β-catenin, and c-Myc while upregulating GSK3β and p-β-catenin. These results suggested that PNS could suppress inflammation, improve intestinal microbiota, and inhibit the Wnt signaling pathway to inhibit CRC development (Fig. 4) .

### EGFR signaling pathway

**Fig. 5:**
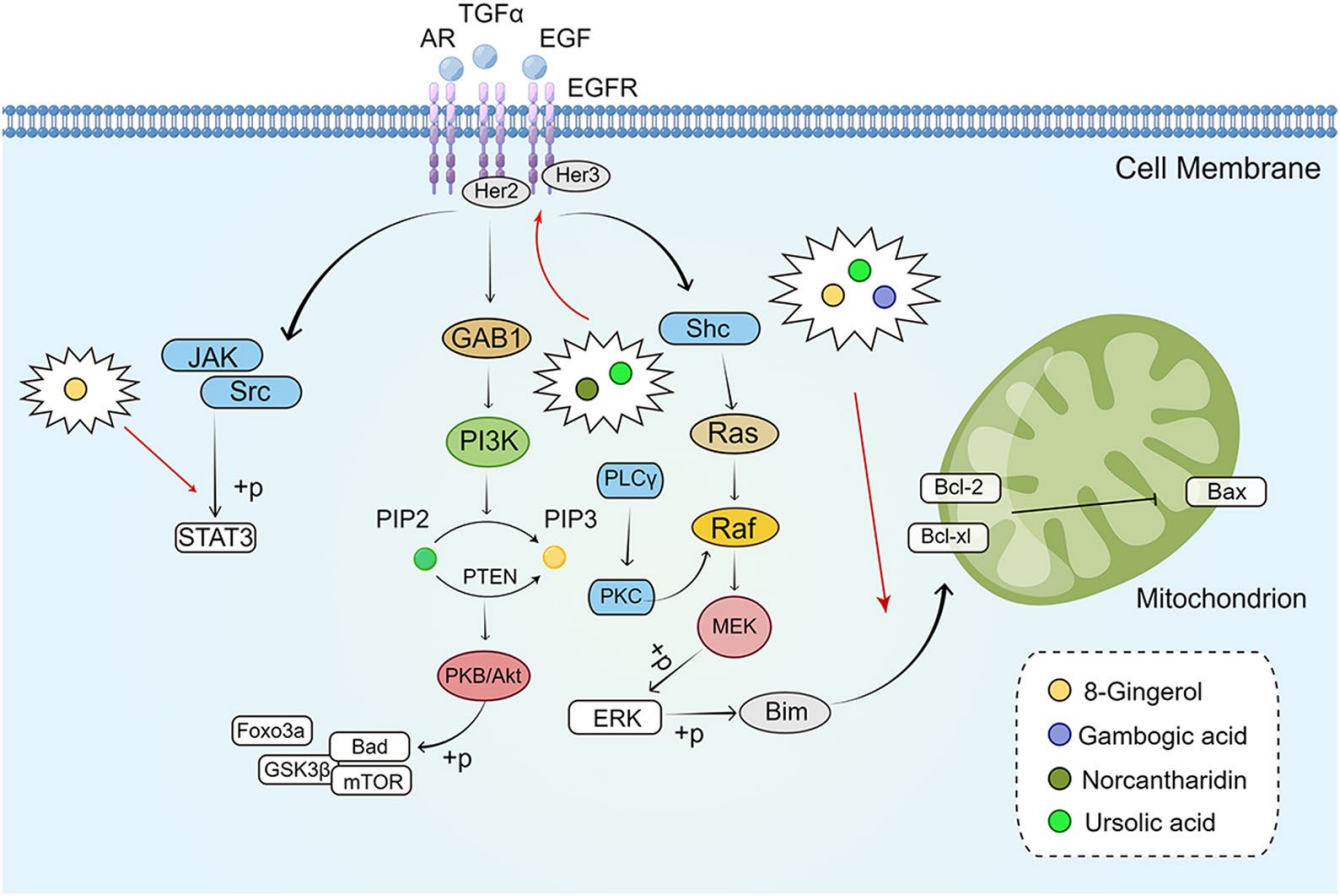
The active compounds of TCM act on the EGFR signaling pathway. The figure was created by Figdraw (https://www.figdraw.com/static/index.html#/)

The EGFR family is composed of four related domains, namely, the extracellular ligand-binding domain, the hydrodynamic transmembrane region, the intracellular RTK domain, and the C-terminal domain, which could assist in participating in a series of physiological activities related to epithelial cells [129–133]. Multiple ligands, including EGF, bind to the receptor and induce its dimerization, thereby activating tyrosine kinase (TK) and multiple downstream effectors [134]. Many ligands, such as am-phiregulin (AR), betacellulin, epidermal growth factor (EGF), heparin-binding EGF-like growth factor, and transforming growth factor α (TGF-α) could activate EGFR [134, 135]. The EGFR phosphorylation response is activated and then signals to downstream pathways, such as the Ras/MAPK pathway, the PI3K/Akt pathway, and the phospholipase C/protein kinase C signaling cascade, which ultimately participate in various cellular activities such as cell survival, proliferation, differentiation, motility, and apoptosis [136, 137]. EGFR and other family members are overexpressed or amplified in cancer, causing uncontrolled proliferation of tumor cells. The receptors are internalized and disrupted upon activation, attenuating the signal or cycling to the outer membrane, resulting in persistent signaling [132, 133]. EGFR is overexpressed in most solid tumors, such as non-small cell lung cancer, head and neck squamous cell carcinoma, CRC, and breast cancer [138–140]. Such tumors are aggressive, drug resistant, and rapidly growing. Therefore, targeting EGFR is one of the directions to develop and design anti-cancer drugs. Currently, the EGFR inhibitors cetuximab and pantitumumab are clinically used to treat mCRC [141, 142] (Fig. 5).

8-Gingerol is one of the main active ingredients of *Zingiber officinale* Rosc (Sheng Jiang) [143]. Hu et al. [144] found that the phosphorylation levels of EGFR and its downstream effectors STAT3 and ERK were reduced in HCT-166 and DLD1 cells after 8-gingerol treatment, which, in turn, led to the downregulation of the expression of target genes, cyclin D1, c-Myc, and MMP2. Meanwhile, the addition of EGF could restore the above phosphorylation levels and protein expression, suggesting that 8-gingerol exerts its anti-proliferative and migratory effects on CRC cells through the EGFR/STAT/ERK signaling pathway. Gambogic acid (GA) is an active component extracted from *Gamboge hanburyi* (Teng Huang), which has been proven to have antitumor effects in many tumors [145–147]. Wei et al. [148] found that the expression of stemness-related genes, such as Nanog, octamer-binding transcription factor 4, SRY-box transcription factor 2 (SOX2), aldehyde dehydrogenase 1, cluster of differentiation 133 (CD133), and B-lymphoma Mo-MLV insertion region 1 (Bmi-1), were significantly reduced in CRC cell lines after GA intervention. The protein expression of its downstream gene zinc-finger protein 36 (ZFP36) was enhanced by inhibiting the phosphorylated expression of EGFR and ERK. The findings suggested that GA could reduce stemness-related genes in CRC cells and exert inhibitory effects on CRC stem cells by suppressing the activation of the EGFR/ERK/ZFP36 signaling pathway. Norcantharidin (NCTD) is an active ingredient isolated and demethylated from *Mylabris phalerata* Pall (Ban Mao), which has significant antitumor activity [149]. Qiu et al. [150] found that NCTD inhibited the expression of EGFR, human epidermal growth factor receptor-2, and c-MET factor and their phosphorylation in HCT-116 and HT-29 cells in a dose-dependent manner; downregulated the expression of cycle-related proteins cyclinD1, Rb, and cyclin-dependent kinase 4; induced G2/M phase block; and promoted apoptosis. In addition, Shan et al. [151] found that ursolic acid (UA), the active ingredient contained in Chinese herbs, such as *Hedyotic diffusa* (Bai Hua She She Cao) and *Radix actinidiae* (Mi Hou Tao), also exerted anti-proliferative and pro-apoptotic effects on HT-29 cells by a mechanism related to activation of caspase-3 and -9; downregulation of Bcl-2 and Bcl-xL protein expression; and inhibition of phosphorylation of EGFR, RK1/2, p38 MAPK, and JNK expression. This finding suggested that UA may exert anti-CRC effects through inhibition of the EGFR/MAPK signaling pathway (Fig. 5).

### p53 signaling pathway

**Fig. 6:**
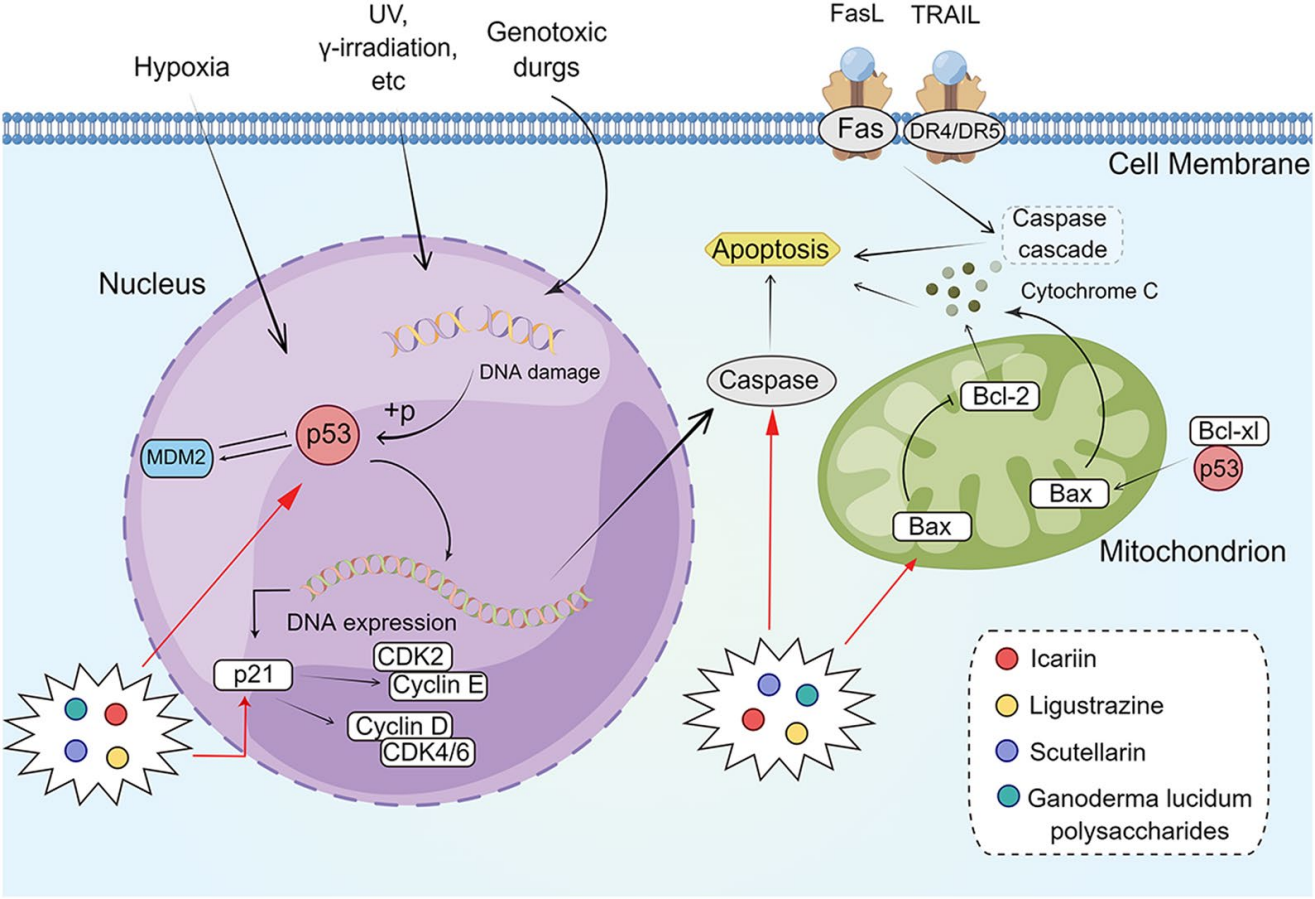
The active compounds of TCM act on the p53 signaling pathway. The figure was created by Figdraw (https://www.figdraw.com/static/index.html#/)

The human p53 gene is located on chromosome 17p, and it consists of 11 exons and 10 introns [152]. As the “guardian of the genome,” p53 could induce cell cycle arrest, apoptosis, or senescence in the presence of cellular stress, thus preventing tumor progression [153]. In normal stress-free cells, the level of p53 remains low. Once p53 is activated, MDM2, an E3 ubiquitin ligase, is upregulated, leading to ubiquitination of p53 and mediation of its degradation, which forms an autoregulatory negative feedback loop [154, 155]. Activation of p53 triggers intrinsic (mitochondria) and extrinsic (death receptors) apoptotic pathways [156]. In the intrinsic pathway, p53 induces the expression of pro-apoptotic Bcl-2 family proteins, such as p53-upregulated modulator of apoptosis and Bax, and downregulates Bcl-2 to trigger permeabilization of the outer mitochondrial membrane. Subsequently, the cytochrome c in the mitochondria is released into the cytoplasm; binds to apoptotic protease activating factor 1; induces activation of promoter caspase-9; and further activates the actuators caspase-3, -6, and -7 [157]. The exogenous pathway mediated by p53 is accompanied by the upregulation of death receptors, such as Fas (CD95/APO-1), DR5 (TRAIL-R2), and p53-induced protein with a death domain, which, together with caspase-8, form a death-inducing signal transduction complex to further activate caspase-3 and induce apoptosis [158]. p53 is the most commonly mutated gene in human cancers [159]. Mutations or loss of function in the p53 gene have been reported in approximately 50–70% of CRC cases [160]. Mutations in p53 not only play a key role in the adenoma-carcinoma transformation of tumors during pathogenesis but also contribute to the aggressiveness of CRC [153, 161]. In addition, p53 interacts with cyclooxygenase-2 to promote inflammation and CRC cell proliferation [162]. Reactivation and restoration of p53 function have great potential in the treatment of CRC (Fig. 6).

Icariin is an active ingredient extracted from *Herba epimedii* (Yin Yang Huo), which has been shown to have antitumor activity against human malignancies [163, 164]. Tian et al. [165] found that the levels of p21, p-p53, and Bax in HCT-116 cells treated with icariin increased, whereas those of Bcl-2 decreased. These results indicated that icariin could induce apoptosis of HCT-116 cells by activating the p53 pathway. Ligustrazine (LZ), the active ingredient in *Ligusticum Chuanxiong* Hort. (Chuan Xiong), has shown antitumor activity in vitro and in vivo, and it is capable of anti-angiogenesis and reversing drug resistance [166–168]. Bian et al. [169] found that LZ could dose-dependently upregulate the levels of p53, Bax, cleaved caspase-3, cleaved caspase-9, and cleaved PARP and downregulate the level of Bcl-2 in SW480 and CT26 cells. Moreover, the expression levels of these proteins were reversed after pretreatment with the p53 inhibitor PFT-α, suggesting that LZ-induced apoptosis of SW480 and CT26 cells was mediated through the p53-dependent mitochondrial pathway. Scutellarin is a flavonoid isolated from *Scutellaria barbata* D. Don (Ban Zhi Lian). Yang et al. [170] found that the expression of Bcl-2, p-p53, and p21 was significantly decreased, whereas that of Bax was significantly increased in HCT-116 cells after scutellarin intervention. These results suggested that the p53 pathway may be involved in scutellarin-induced apoptosis of HCT-116 cells. In addition, *Ganoderma lucidum* polysaccharides (GLPs), which are isolated from spores, mycelia, and fruiting bodies of *G. lucidum* (Ling Zhi), showed anticancer effects [171, 172]. Jiang et al. [173] found that HCT-116 cells transfected with mutant p53R273H and p53248W showed upregulated expression of Bax and p21 and underwent G1 phase cell-cycle arrest and apoptosis after combined treatment of GLPs and GLPs combined with 5-FU. These findings showed that GLPs exert their effects on inducing growth inhibition and apoptosis in CRC cells through reactivation of p53 (Fig. 6).

### TGF-β/Smad signaling pathway

**Fig. 7:**
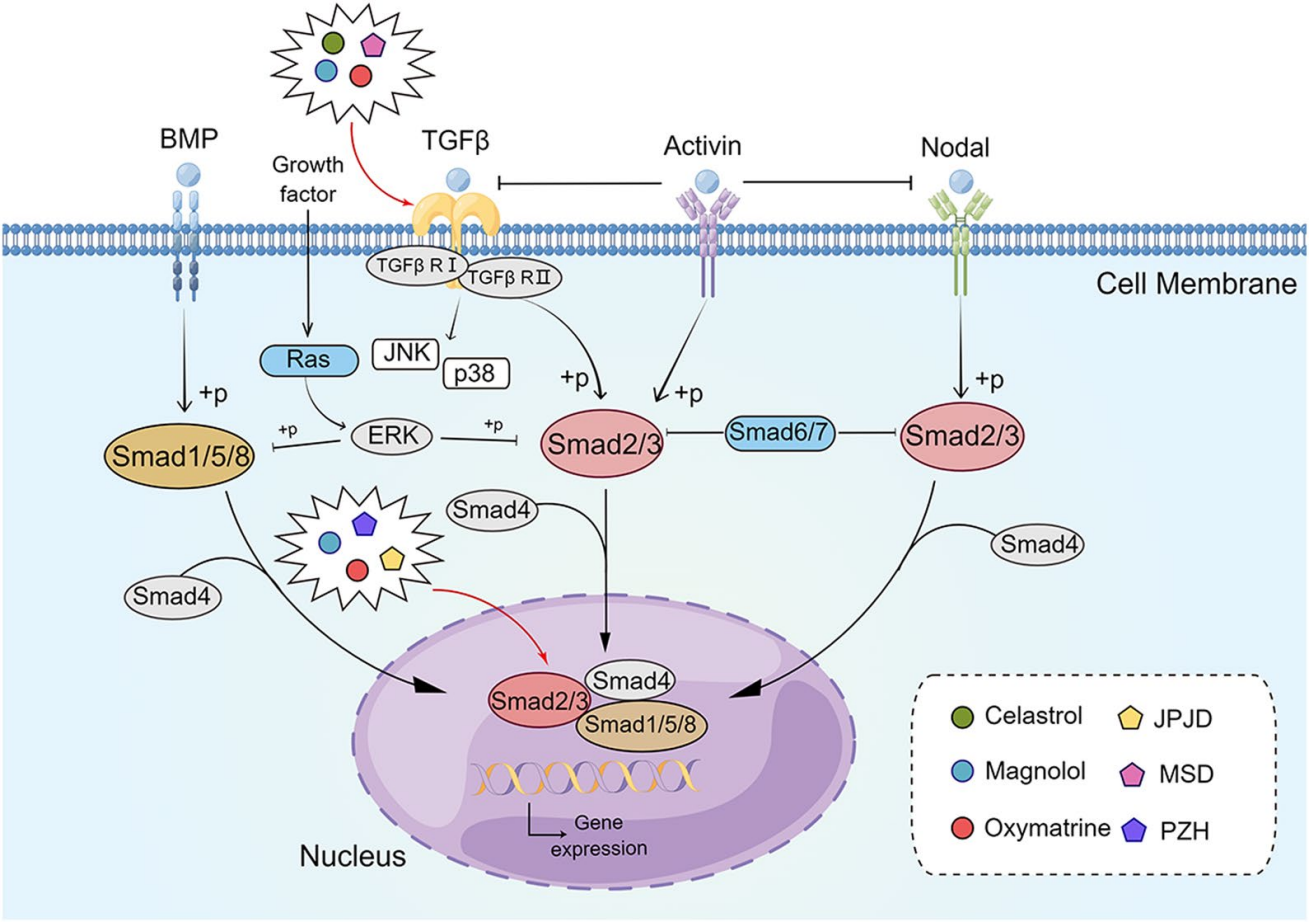
The active compounds of TCM and the Chinese herb formula act on the TGF-β/Smad signaling pathway. *JPJD* JianPi JieDu Recipe, *MSD* modified Shenlingbaizhu Decoction, *PZH* Pien Tze Huang. The figure was created by Figdraw (https://www.figdraw.com/static/index.html#/)

The transforming growth factor-β (TGF-β) family proteins include TGF-β1, -β2, and -β3; activins; nodal; bone morphogenetic proteins; and growth proteins and differentiation factors [174]. As a multifunctional cytokine, TGF-β exerts pleiotropic effects on cell physiology, such as proliferation, survival, differentiation, and migration [175]. SMAD proteins are key intracellular signaling mediators and transcription factors for TGF-β superfamily signaling [176]. TGF signaling is initiated by the TGF ligand binding to the type II TGF receptor (TGFBR2). Upon binding to the ligand, TGFBR2 recruits and phosphorylates the type I TGF-β receptor, which, in turn, initiates downstream signaling by phosphorylating the transcription factors SMAD2 and SMAD3 [177] (Fig. 7).

TGF-β plays a double-edged role in the progression of tumors. In normal epithelial cells and early tumor cells, TGF-β increased the expression of CDK inhibitors p15INK4, p21CIP1, p27KIP1, and p57KIP2 via the canonical SMAD pathway to block cell cycle progression [178, 179]. However, in tumor cells, TGF-β promotes tumor progression through mechanisms, such as induction of EMT [180, 181]. The core components of the TGF-β pathway exhibit high levels of loss-of-function mutations in gastrointestinal tumors. In CRC, this phenomenon was mainly manifested by mutations in TGFBR2, SMAD2, and SMAD4, which suppressed the tumor inhibitory ability of TFG-β [182, 183]. In addition, TGF-β receptor could induce other signaling, such as MAPK, PI3K/Akt, Janus kinase-signal transducer, and STAT [184] (Fig. 7).

Celatrol is an effective active ingredient in *T. wilfordii* Hook F. (Lei Gong Teng), which has anti-inflammatory and anticancer effects [185]. Jiang et al. [186] found that celastrol treatment significantly inhibited the mRNA and protein levels of TGF-β1, TGF-βRI, and TGF-βRII in HCT-116 and SW620 cells. It also inhibited Smad signaling and decreased the expression of p-Smad2/3 and Smad4. These results suggested that the TFG-β/Smad signaling pathway is involved in celastrol-mediated anti-CRC effects. Magnolol is an active ingredient extracted from the bark of *Houpu magnolia* (*Magnolia officinalis*) (Hou Pu), and it has a wide range of biological activities [187–189]. Chei et al. [190] discovered that in magnolol-treated HCT-116 cells, the expression of epithelial markers, such as E-cadherin, zona occludens 1 (ZO-1), and claudin, increased in a concentration-dependent manner, whereas that of mesenchymal markers, such as N-cadherin, TWIST1, Slug, and Snail, decreased in a concentration-dependent manner. The expression of p-ERK, p-GSK3β, and p-Smad, the downstream proteins of TGF-β signaling pathway, decreased. These results suggested that magnolol inhibits TGF-β-induced EMT by blocking signal transduction downstream of TGF-β signaling. Oxymatrine (OM), the active ingredient in *Sophora flavescens* Alt. (Ku Shen), could inhibit the growth of various types of tumor cells [191, 192]. Wang et al. [193] found that the expression of E-cadherin was upregulated in RKO cells after OM intervention. On the contrary, the expression of α-smooth muscle actin (α-SMA), fibronectin, TGF-β1, plasminogen activator inhibitor-1 (PAI-1), Smad2/3/4, p-Smad2, and p38 was downregulated, implying that OM could inhibit the TGF-β pathway activation and EMT in CRC by reducing the p38-dependent increase in PAI-1 expression (Fig. 7).

Herbal compounds could also exert anti-CRC effects through the TGF-β/Smad pathway. Liu et al. [194] demonstrated that Jianppi Jieu Recipe (JPJD) could downregulate the expression of p-Smad2/3 and Snail and upregulate the expression of E-cadherin in TGF-β-stimulated Lovo cells in vitro. In-vivo experiments showed that JPJD could upregulate the expression of E-cadherin and downregulate the levels of p-Smad2/3 and Snail in orthotopic CRC tumor tissues in nude mice, suggesting that JPJD may inhibit TGF-β-induced EMT via the expression of Snali/E-cadherin mediated by the TGF-β/Smad2/3 signaling pathway, thus exerting anti-CRC invasive and metastatic effects. Dai et al. [195] found that modified Shenlingbaizhu Decoction (MSD) treatment downregulated the expression of TβRI, CD133, Vimentin, Oct-4 A, and SOX2 in mouse tumor tissues. After the use of TGF-β inhibitor, EMT signal transduction was inhibited, and the pluripotency of colorectal cancer stem cells (CSCs) was reduced. These results suggested that MSD restrains the pluripotency of CSCs by suppressing TGF-β/Smad signaling-induced EMT in vivo while inhibiting the proliferation, migration, and invasion of CRC cells. In addition, Pien Tze Huang could reverse tumor drug resistance by inhibiting the phosphorylation of N-cadherin, TGF-β, Smas2/3, and Smad 4 in tumor tissues while promoting the expression of E-cadherin, thereby inhibiting the movement, invasion, and EMT of CRC cells [196] (Fig. 7).

### mTOR signaling pathway

**Fig. 8:**
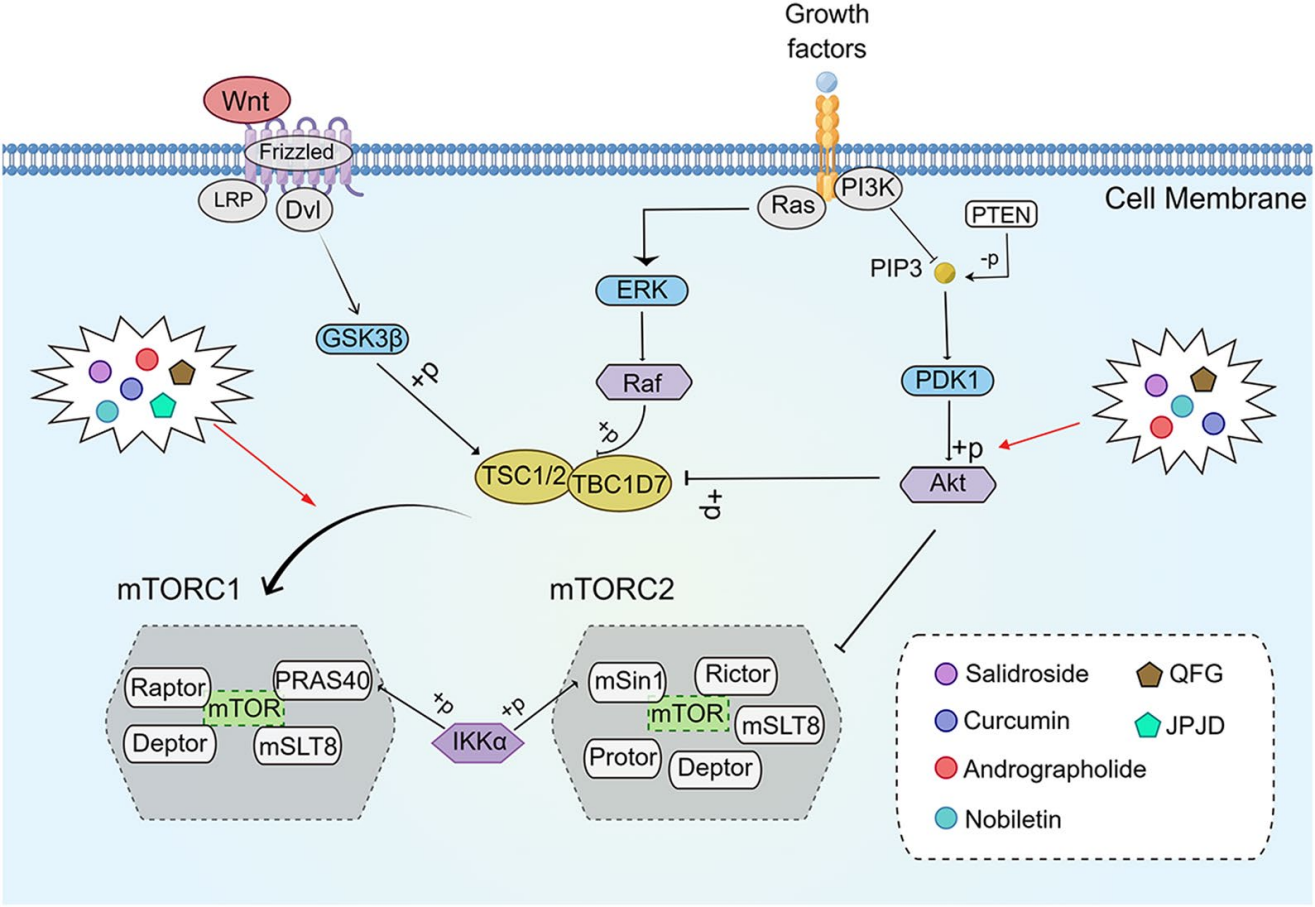
The active compounds of TCM and the Chinese herb formula act on the mTOR signaling pathway. *QFG* Qingjie Fuzheng granule, *JPJD* Jianpi Jiedu Decoction. The figure was created by Figdraw (https://www.figdraw.com/static/index.html#/)

mTOR belongs to the PI3K-related kinase family, a 289 kDa serine/threonine protein kinase containing 2550 amino acids and encoded by 7650 nucleotides [197, 198]. As a major regulator of cell growth and metabolism, mTOR not only promotes the anabolic processes of ribosomes, proteins, nucleotides, fatty acids, and lipids but also inhibits catabolic processes, such as autophagy [199]. In mammals, mTOR contains two major protein complexes with different functions: mTOR complex1 (mTORC1) and mTOR complex2 (mTORC2) [200]. mTORC1 contains mTOR, raptor, mLST8, and two negative regulators, PRAS40 and DEPTOR; it is sensitive to rapamycin [201–204]. mTORC2 consists of mTOR, rictor, mLST8, mSin1, and the newly identified components Protor, Hsp70, and DEPTOR; it is insensitive to rapamycin treatment [205–208]. mTOR signaling has been reported to be overactivated in most human cancers, especially in relation to multiple biological processes, such as cell proliferation, survival, autophagy, metabolism, and immunity [199, 209, 210] (Fig. 8).

The mTOR signaling pathway may have a direct effect on the carcinogenesis of CRC. Elevated RNA and protein levels of mTORA, raptor, and rictor could be observed in the tissues of patients with CRC, and a correlation was found between the degree of elevated levels of the above expression and the stage of malignancy [211, 212]. Elevated mTORC1 and mTORC2 activities were also associated with EMT and RhoA and Rac1 signaling-mediated CRC metastasis, and it was related to chemoresistance [213]. The anti-CRC effects of currently developed mTOR inhibitors have been demonstrated in in-vivo and -vitro assays [214, 215]. Therefore, targeting the mTOR signaling pathway could benefit CRC treatment (Fig. 8).

Salidroside, the active ingredient in *Rhodiola rosea* L. (Hong Jing Tian), has been reported to have significant antitumor effects, exerting antiproliferative and pro-apoptotic effects on many types of malignant tumors [216–218]. Fan et al. [219] found that LC3-II/LC3-I and Beclin-1 expression were increased and Bcl-2/Bax, p-PI3K, p-AKT, and p-mTOR expressed were decreased in HT-29 cells after salidroside treatment. The above changes were reversed after the use of autophagy inhibitor and PI3K inhibitor. The findings suggested that salidroside may exert its pro-apoptotic and autophagic effects on HT-29 cells by inhibiting the PI3K/Akt/mTOR signaling pathway. Curcumin is the active ingredient of *R. Curcumae* (Jiang Huang) [220]. Johnson et al. [221] found that curcumin induced Akt phosphorylation in HCT-116 cells while decreasing the expression of mTOR, raptor, and rictor proteins, suggesting that curcumin may exert its anti-proliferative effects on CRC cells through the Akt/mTOR signaling pathway. In addition, andrographolide, the active ingredient in *Andrographis paniculata* (Chuan Xin Lian), downregulated the expression of PI3K/p110, p-Akt, p-mTOR, and glycolysis-related proteins, such as phosphofructokinase 1, GLUT1, and hexokinase 2, in HCT-116 cells, suggesting that andrographolide may enhance radiosensitivity by inhibiting glycolysis in HCT-116 cells via the PI3K/Akt/mTOR signaling pathway [222]. Nobiletin, an active ingredient in *Citrus reticulata* Blanco (Gan Ju), has biological effects such as anti-inflammatory, anti-cancer, and neuroprotective effects [223, 224]. Li et al. [225] found that nobiletin could enhance the inhibitory effect of oxaliplatin on the proliferation of HT29 and SW480 cells, while upregulating the expression of pro-apoptotic proteins Bax and caspase3, and downregulating the expression of p-Akt, p-mTOR and anti-apoptotic protein Bcl-2 to promote oxaliplatin-induced apoptosis. The findings above suggested that nobiletin may enhance CRC sensitivity to oxaliplatin by downregulating the PI3K/Akt/mTOR signaling pathway.(Fig. 8).

Zhu et al. [226] found that the Chinese herbal formulation Qingjie Fuzheng granule (QFG) could not only upregulate the expression of E-cadherin, LC3-II, and Beclin-1 but also downregulate the expression of N-cadherin, Vimentin, TWIST1, and p62 in HCT-116 xenograft tumors. Moreover, the ratios of PI3K/PI3K, p-AKT/AKT, and p-mTOR/mTOR were significantly lower than those in the controls, suggesting that QFG may induce CRC autophagy and thus inhibit EMT progression through the mTOR signaling pathway. Peng et al. [227] found that Jianpi Jiedu Decoction (JPJD) downregulated the expression of mTOR, HIF-1α, VEGF, phosphorylation ribosomal protein S6 kinase (p-p70S6K), and phosphorylation 4E binding protein 1 (p-4E-BP1) in vivo and in vitro, indicating that JPJD regulates the mTOR/HIF-1α/VEGF signaling pathway to exert antitumor activity (Fig. 8).

### Hedgehog signaling pathway

**Fig. 9:**
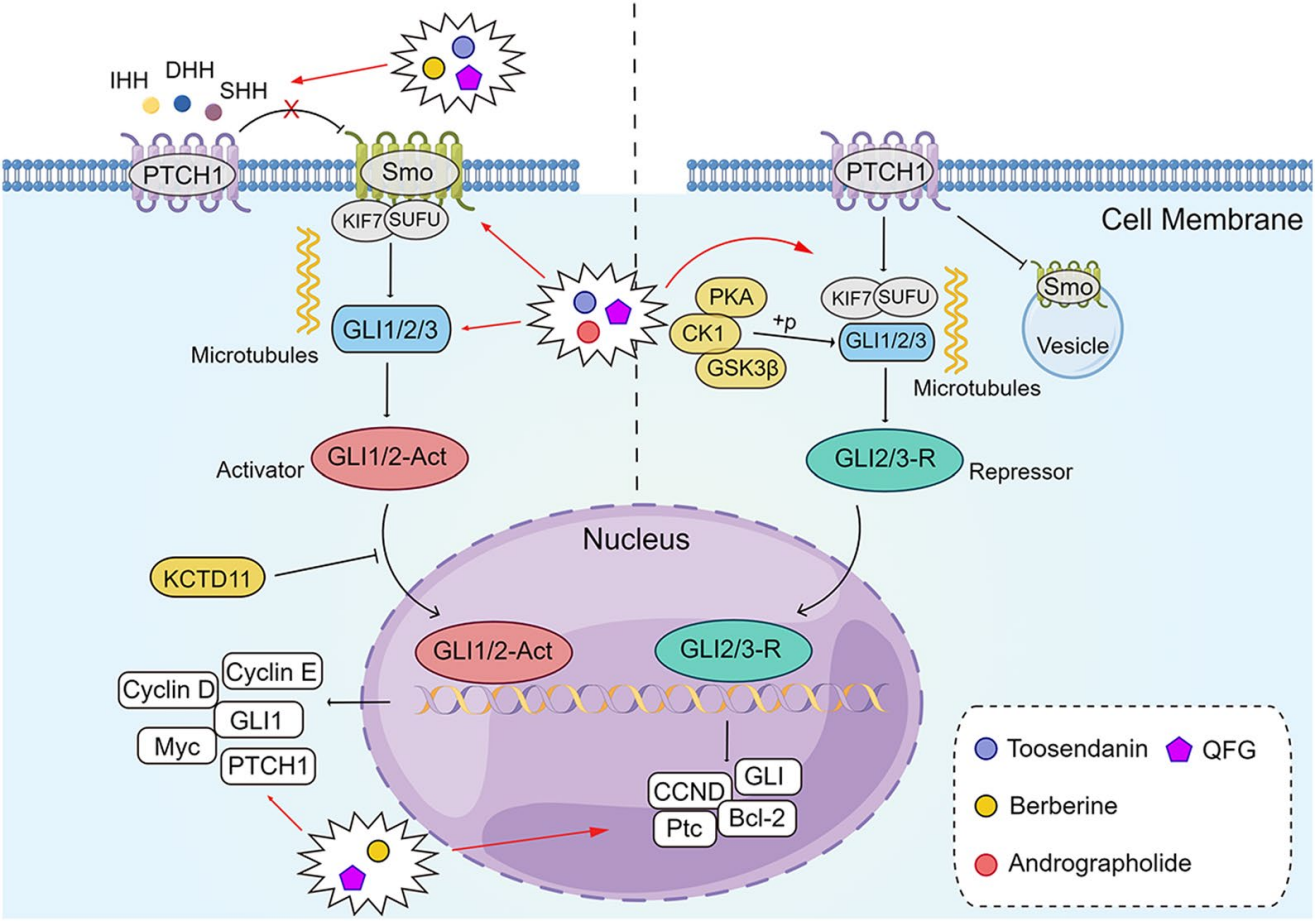
The active compounds of TCM and the Chinese herb formula act on the Hedgehog signaling pathway. *QFG* Qingjie Fuzheng granule. The figure was created by Figdraw (https://www.figdraw.com/static/index.html#/)

In mammals, three ligands of Hedgehog (HH) exist, namely Indian Hedgehog (IHH), Sonic Hedgehog (SHH) and Desert Hedgehog (DHH), capable of participating in the patterning and development of various tissues and organs [228, 229]. Both IHH and SHH can expressed in the gastrointestinal tract, whereas DHH appears to be expressed only in the nervous system and testis [230]. The major components of the HH pathway are located in the cell membrane [231]. The transduction response to HH ligands is mainly regulated and transmitted by two transmembrane proteins: patched (Ptc) and smoothened (Smo), and downstream transcription factors of the Gli family (Gli1, Gli2, and Gli3) [232]. In the absence of its ligand, the HH receptor patched 1 (PTCH1) inhibits Smo function by preventing Smo from entering primary cilia. When HH ligands bind to Ptc, this mutual inhibition is relieved and Smo signaling is activated [233, 234]. The Smo-repressing activity of Ptc is inhibited by binding to HH, thereby releasing Smo and exhibiting its signaling activity within the cell. Smo, located in the primary cilia, signals intracellularly to mediate three Gli zinc finger transcription factors [235]. The transcription factors Gli1, Gli2, and Gli3 are the major downstream executors of HH activation and the key final outputs of HH. Gli1 is an HH-responsive gene product that acts only as a transcriptional activator and participates in a positive feedback loop during pathway activation, while Gli2 and Gli3 act as the main transcriptional activator and transcriptional inhibitor, respectively [236]. Inappropriate activation of the Hedgehog signaling pathway is common in various tumors, such as pancreatic cancer, breast cancer and gastric cancer [237, 238] (Fig. 9).

Numerous studies have shown that the Hedgehog signaling pathway is involved in CRC tumorigenesis. The SHH pathway has a facilitative role in angiogenesis, cell proliferation, and metastasis, while downregulation of IHH has been observed to be an early event in CRC formation [239–241]. In addition, the coordination of Smo and Gli, the downstream components of the Hedgehog signaling pathway, plays the most important role in HH regulation of CRC [242]. Cyclopamine, an HH inhibitor, has been demonstrated to anti-proliferation and pro-apoptosis effects in CRC cells [243, 244]. Therefore, regulating the Hedgehog signaling pathway also offers an approach to treating CRC (Fig. 9).

Toosendanin (TSN), an active component derived from *MeLia toosendan* Sieb. et Zucc. (Chuan Lian Zi), has anti-cancer properties in malignant tumors such pancreatic cancer, gastric cancer, and osteosarcoma [245–248]. Zhang et al. [249] found that TSN could down-regulate the mRNA and protein levels of SHH, GLI1 and SMO in HT-29 cells in a concentration-dependent manner and inhibit the proliferation of HT-29 cells in vitro. In vivo, it was able to reduce the weight of HT-29 xenograft tumors, meanwhile the protein expression levels of SHH in TSN-treated tumor tissues were significantly lower than those in the control group. These findings implied that TSN may inhibit growth of CRC cells by inhibiting the Hedgehog pathway, and the target of TSN may be SHH. Sun et al. [250] discovered that following Berberine (BBR) treatment, the expression of SHH, Ptch1, SMO, Gli1, and c-Myc was down-regulated while the expression of SUFU was up-regulated in HT-29 cells, suggesting that BBR exerts anti-CRC effects by decreasing the Hedgehog signaling pathway activity. Khan et al. [251] found that andrographolide, the active ingredient in *Andrographis paniculata* (Chuan Xin Lian), had a strong cytotoxic effect on HCT-116 cells, induced G2/M phase block by downregulating the expression of CDK1 and CyclinB1, downregulated the mRNA levels of Gli1 and SMO, and had a high affinity for the Smo protein. These results indicated that andrographolide may exert anti-CRC effects by suppressing the Hedgehog signaling pathway. Furthermore, Qingjie Fuzheng Granule (QFG) could significantly reduce the protein expression of the Sonic Hedgehog pathway-related proteins SHH, Smo and Gli1 in HCT-116 xenograft mice tumor tissues. It also down-regulated the expression of pro-proliferative survivin Ki-67, CyclinD1, CDK4, VEGF-A and VEGFR-2, and up-regulated the ratio of Bax/Bcl-2 and the expression of p21. These results suggested that QFG exerts anti-CRC effects by inhibiting the Sonic Hedgehog pathway to prevent CRC cells from proliferation, promoting apoptosis and anti-tumor angiogenesis [252] (Fig. 9).

### The immunomodulatory signaling pathways

The development of tumors is significantly influenced by the immune system. Tumor incidence rises as the host’s immune system performs worse. The tumor microenvironment (TME), which is strongly associated to tumor invasion and metastasis and encourages tumor progression, is made up of tumor cells, immune cells, extracellular matrix, and interstitial tissue [253]. Cancer immunotherapy is one of the new options for cancer treatment. In contrast to standard treatments such as surgery, radiation and chemotherapy, immunotherapy could effectively overcome the specificity problems associated with radiation and chemotherapy by using and manipulating the patient’s own immune system to fight and destroy tumor cells [254]. Cytokines such as interleukin-2 (IL-2), interferon-γ (IFN-γ), interleukin-6 (IL-6), and TNF-α play an important role in regulating immune responses and anticancer defense systems [255, 256]. The suppression of immune function and the imbalance of cytokines are the key factors leading to the occurrence and development of cancer and the poor prognosis of patients. Furthermore, immune evasion is one of the hallmarks of tumors. Although natural killer (NK) cells are able to recognize and kill tumor cells, tumor cells from TME are able to inhibit the activity of NK cells [257]. High levels of CD8^+^ CTL, and CD4^+^ helper T cells (Th cells) are favorable prognostic factors, while elevated levels of CD4^+^Tregs indicate a poor prognosis [258]. M1 and M2 are the two main stages of macrophages. T helper (Th-1) cytokines could activate macrophage M1, while Th-2 cytokines such as IL-4 could selectively activate macrophage M2 [259]. Tumor-associated macrophages (TAMs) with the M2 phenotype are important regulators in the occurrence and development of cancer, which can lead to a poor prognosis of cancer patients [260]. Studies have confirmed that immune checkpoint inhibitors (ICIs) can reactivate the immune system and kill tumor cells [261].

TCM has shown potential as ICIs in the treatment of malignant tumors. Unlike Western medicine, which mainly directly kills tumor cells, TCM can systematically regulate TME and exert anti-CRC effects through immunomodulatory signaling pathways [262]. *Ganoderma atrum* polysaccharide (PSG-1) is a polysaccharide component isolated from the Chinese herb *G. lucidum* (Ling Zhi) [263]. Zhang et al. [264] found that PSG-1 had no anti-tumor activity in vitro, but was effective in activating peritoneal macrophages in CT26 tumor-bearing mice, enhancing the phagocytic capacity of macrophages and promoting the production of nitric oxide (NO), TNF-α and IL-1β in mice to enhance immune function. Furthermore, it was discovered that PSG-1 acted on toll-like recepetor 4 (TLR4) and then activated NF-κB via the p38 MAPK pathway, promoting TNF-α production, IκBα degradation, and p38 MAPK phosphorylation. The above results suggested that PSG-1 could activate macrophages through TLR4-dependent signaling pathways to enhance immune function and inhibit tumor growth. *Panax notoginseng* saponins (PNS), the active ingredients extracted from *Panax notoginseng* (Burk) F.H.Chen (San Qi), have shown anti-tumor effects on CRC cells when used alone or in combination with chemotherapeutic drugs [265, 266]. Li et al. [267] found that PNS had significant preventive and immunomodulatory effects on AOM/DSS-induced CAC mice, and was able to alleviate the immunosuppression of Treg cells in the colonic tissue microenvironment of CAC mice by inhibiting signal transduction and direct mediation of indoleamine 2,3-dioxygenase 1 (IDO1) expression by transcription 1 (STAT1), reducing macrophage aggregation, and reshaping the CAC immune microenvironment. Genkwanin is the active ingredient in *Daphne genkwa* Sieb. et Zucc. (Yuan Hua). Wang et al. [268] found that genkwanin effectively inhibited the proliferation of HT29 and SW480 cells and the secretion of inflammatory factor IL-8 in vitro, increased the spleen and thymus indices in APC^Min/+^ mice in vivo, and effectively reduced the levels of IL-1α, IL-1β, IL-8, G-CSF and GM-CSF in mouse colon tissues. These findings suggested that genkwanin has antitumor activity, most likely by enhancing the immune response and decreasing the levels of inflammatory factors.

## Conclusion

Intracellular signaling pathways are involved in various biological processes, and they are closely associated with the progression of CRC. TCM has a long history of development and plays an important role in the prevention and treatment of malignant tumors with its unique dialectical concept and holistic concept. In recent years, many basic experiments and clinical studies have confirmed that TCM has a good effect in the treatment of CRC, which can effectively improve the immune function of CRC patients, improve the quality of life, and prolong the survival time [269–271]. In this paper, the mechanisms by which TCM exert anti-CRC effects were summarized from the perspective of signaling pathways. TCM could regulate multiple signaling pathways related to CRC progression, including PI3K/Akt, NF-κB, MAPK, Wnt/β-catenin, EGFR, p53, TGF-β, mTOR, Hedgehog, and immunomodulatory signaling pathways, thus affecting biological processes, such as cell proliferation, apoptosis, cell cycle, migration, invasion, autophagy, EMT, angiogenesis, and chemoresistance and ultimately exerting therapeutic effects on CRC (Tables 1 and 2). Given the complexity of CRC pathogenesis, TCM with multiple components, targets, and effects is expected to be a breakthrough in the development of therapeutic CRC drugs. As study on the theory and practice of TCM continues to advance, the avenues and means by which TCM exerts its healing effects could be further elucidated.

**Table 1:** Effects of TCM and its active ingredients on preventing and treating CRC

Active ingredients	Herb source	Experiments		Effects	Molecular mechanism	Signal pathways	Refs.
		In vitro model	In vivo model				
Wogonin (WOG)	Huang Qin (*Scutellaria baicalensis* Georgi)	SW48 cells	–	Autophagy ↑, Apoptosis ↑, Block cell cycle	↑: LC3II, Beclin-1, caspase-3, caspase-8, caspase-9, Bax ↓: Bcl-2, p-PI3K, p-Akt	PI3K/Akt STAT3	[44]
Coptisine (COP)	Huang Lian (*Coptis chinensis* Franch.)	HCT-116	–	Apoptosis ↑, Block cell cycle	↑: cleaved caspase-3, cleaved caspase-8 ↓: PI3K,Akt, ERK, CyclinD1, CyclinE, CDK2, CDK4	PI3K/Akt ERK	[47]
		–	HCT-116 xenograft mice	Tumor growth ↓	↓: CEA, CA19-9, CYFRA 21 − 1		
Emodin (EMD)	Da Huang (*Rheum palmatum* L.)	HCT-116	HCT-116 xenograft mice	Cell growth ↓, Adhesion ↓, Migration ↓	↓: VEGFR2, PI3K, p-Akt	VEGFR2/PI3K/Akt	[49]
Triptolide (TP)	Lei Gong Teng (*Tripterygium wilfordii* Hook. f.)	HT29、SW480	–	Proliferation ↓	↓: p-Akt	Akt	[50]
Platycodin D (PD)	Jie Geng (*Platycodon grandiflorus* (Jacq.) A. DC.)	HCT-116、LoVo	HCT-116 and LoVo subcutaneous tumor mice	Proliferation ↓, Migration ↓,Invasion ↓	↓: p-PI3K, p-Akt	PI3K/Akt	[54]
Oridonin (ORI)	Dong Ling Cao (*Rabdosia rubescens*( Hemsl.) Hara)	LoVo、SW480	–	Proliferation ↓, Apoptosis ↑	↑: HERC5 ↓: AP-1, NF-κB, P38	NF-κB P38-dependent MAPK	[71]
		–	LoVo、SW480 Colostomy implantation model mice	Tumor growth ↓	↓: AP-1, NF-κB, P38		
Baicalein (BE)	Huang Qin (*Scutellaria baicalensis* Georgi)	HCT-116	–	Apoptosis ↑, Migration ↓, Invasion ↓	↑: PPARγ ↓: caspase-3, caspase-,, MMP2, MMP, p50, P65, iNOS	NF-κB	[73]
Berberine (BBR)	Huang Lian (*Coptis chinensis* Franch.), Huang Bai (*Phellodendron chinense* Schneid.), Xiao Bo (*Berberis silva-taroucana* Schneid.)	–	AOM/DSS induced CRC model mice	Proliferation ↓, inflammation ↓, Angiogenesis ↓, Invasion ↓	↓: Ki-67, β-catenin, IL-1b, TNF-α, NF-κB, MMP9, Ereg, Muc16	NF-κB	[74]
Curcumin	Jiang Huang (*Curcuma longa* L.)	HCT-116、SW620、HT-29	HCT-116 orthotopic mice	Proliferation ↓, Invasion ↓, Angiogenesis ↓, Apoptosis ↑	↓: NF-κB, COX2, Cyclin D1, c-myc, MMP9, ICAM-1, CXCR4, survivin, Bcl-2, VEGF	NF-κB	[75]
Ginsenoside Rh1 (Rh1)	Ren Shen (*Panax ginseng* C.A.Mey.)	SW620	–	Proliferation ↓, Migration ↓, Invasion ↓	↑: TIMP3 ↓: MMP1, MMP3, p-P38/P38, p-ERK1/2/ERK1-2, p-JNK/JNK	MAPK	[94]
		–	SW620 xenograft mice	Tumor growth ↓	↓: p-P38/P38, p-ERK1/2/ERK1-2, p-JNK/JNK		
Diterpenoid C	Jiang Huang (*Curcuma longa* L.)	SW620	–	Proliferation ↓, Apoptosis ↑	↑: caspase-3 ↓: p-ERK, p-JNK, p-p38 MAPK	MAPK	[96]
Podophyllotoxin (PT)	Gui Jiu (*Podophyllum peltatum*)	HCT-116	–	Apoptosis ↑, Block cell cycle	↑: ROS, GRP78, CHOP, p38 MAPK	p38 MAPK	[100]
Emodin (EMD)	Da Huang (*Rheum palmatum* L.)	SW620、SW480	–	Apoptosis ↑, Migration ↓, Invasion ↓	↑: HBP1 ↓: CTNNB1, TCF7L2, cyclin D1, c-Myc, snail, vimentin, MMP-2, MMP-9, p300	Wnt/β-catenin	[117]
Ginkgolide C (GGC)	Yin Xing Ye (*Ginkgo biloba* L.)	HT-29	–	Proliferation ↓, Apoptosis ↑, Migration ↓, Invasion ↓	↓: MMP2, MMP9, Wnt3a, β-catenin, Axin-1,p-GSK3β, β-TrCP, c-myc, cyclin D1, survivin	Wnt/β-catenin	[120]
Curcumin	Jiang Huang (*Curcuma longa* L.)	SW620	–	Proliferation ↓, Apoptosis ↑	↑: CDX2 ↓: Wnt3a, c-Myc, survivin, cyclin D1	CDX2/Wnt/β-catenin	[124]
8-Gingerol	Sheng Jiang (*Zingiber officinale* Rosc.)	HCT-116、DLD1	–	Proliferation ↓, Migration ↓	↓: p-EGFR, p-STAT, p-ERK, cyclin DI, c-Myc, MMP2	EGFR/STAT/ERK	[144]
Gambogic acid (GA)	Teng Huang (*Garcinia hanburyi* Hook.f.)	HCT-116、SW480	–	Growth ↓, EMT ↓	↑: N-cadherin, vimentin, fibronectin ↓: Nanog, Oct4,Sox2, ALDH1, CD133, Bmi-1, E-cadherin, p-EGFR, p-ERK, ZFP36	EGFR/ERK/ZFP36	[148]
Norcantharidin (NCTD)	Ban Mao (*Mylabris phalerata* Pallas)	HCT-116、HT-29	–	Apoptosis ↑, Block cell cycle	↓: EGFR, p-EGFR, Her-2, p-hER-2, c-Met, p-c-Met, CyclinD1, Rb, CDK-4	EGFR	[150]
Ursolic acid (UA)	Bai Hua She She Cao (*Oldenlandia diffusa* (willd.) Roxb), Mi Hou Tao (*Actinidia chinensis* Planch.)	HT-29	–	Proliferation ↓, Apoptosis ↑	↑: caspase-3, caspase-9 ↓: Bcl-2, Bcl-xL, p-EGFR, p-RK1/2, p-p38 MAPK, p-JNK	EGFR/MAPK	[151]
Icariin	Yin Yang Huo (E*pimedium brevicornum* Maxim.)	HCT-116	-	Apoptosis ↑	↑: p21, p-p53, Bax ↓: Bcl-2	p53	[165]
Ligustrazine (LZ)	Chuan Xiong (*Ligusticum chuanxiong* Hort.)	SW480, CT26	–	Apoptosis ↑, Block cell cycle	↑: p53, Bax, cleaved caspase-3, cleaved caspase-9, cleaved PARP ↓: Bcl-2	p53	[169]
Scutellarin	Ban Zhi Lian (*Scutellaria barbata* D. Don)	HCT-116	–	Apoptosis ↑	↑: Bax ↓: Bcl-2, p-p53, p21	p53	[170]
Polysaccharides (GLPs)	Ling Zhi (*Ganoderma lucidum* (Leyss.ex Fr.) Karst.)	HCT-116	–	Growth ↓, Apoptosis ↑, Block cell cycle	↑: p21, Bax	p53	[173]
Celastrol	Lei Gong Teng (*Tripterygium wilfordii* Hook. f.)	HCT-116, SW620	–	Propagation ↓, Adhesion ↓, Metastasis ↓	↓: TGF-β1, TGF-βRI, TGF-βRII, p-Smad2/3, Smad4	TGF-β/Smad	[186]
Magnolol	Hou Po (*Magnolia officinalis* Rehd.et Wils.)	HCT-116	–	EMT ↓	↑: E-cadherin, ZO-1, claudin ↓: N-cadherin, TWIST1, Slug, Snail, p-ERK, p-GSK3β, p-Smad	TGF-β	[190]
Oxymatrine (OM)	Ku Shen (*Sophora flavescens* Ait.)	RKO	–	EMT ↓	↑: E-cadherin ↓: α-SMA, FN, TGF-β1, PAI-1, Smad2/3/4, p-Smad2, P38	TGF-β/Smad	[193]
Salidroside	Hong Jing Tian (*Rhodiola rosea* L.)	HT-29	–	Apoptosis ↑, Autophagy ↑	↑: LC3-II/LC3-I, Beclin-1 ↓: Bcl-2/Bax, p-PI3K, p-AKT, p-mTOR	PI3K/Akt/mTOR	[219]
Curcumin	Jiang Huang (*Curcuma longa* L.)	HCT-116	–	Proliferation ↓	↑: p-Akt ↓: mTOR, Raptor, Rictor	Akt/mTOR	[221]
Andrographolide	Chuan Xin Lian (*Andrographis paniculata* (Burm. F.) Nees)	HCT-116	–	Glycolysis ↓	↓: PI3K/p110, p-Akt, p-mTOR, PFK1, GLUT1, HK2	PI3K/Akt/mTOR	[222]
Nobiletin	Gan Ju (*Citrus reticulata* Blanco)	HT-29、SW480	–	Tumor growth ↓, Immunity ↑	↑: Bax, caspase3 ↓: p-Akt, p-mTOR, Bcl-2	PI3K/Akt/mTOR	[225]
Toosendanin (TSN)	Chuan Lian Zi (*MeLia toosendan* Sieb.et Zucc.)	HT-29	HT-29 xenograft nude mice	Proliferation ↓	↓: SHH, GLI1, SMO	Hedgehog	[249]
Berberine (BBR)	Huang Lian (*Coptis chinensis* Franch.), Huang Bai (*Phellodendron chinense* Schneid.), Xiao Bo (*Berberis silva-taroucana* Schneid.)	HT-29	–	Proliferation ↓, Migration ↓, Invasion ↓, Apoptosis ↑	↑: SUFU ↓: SHH, Pthc1, Gli1, SMO, c-Myc	Hedgehog	[250]
Andrographolide	Chuan Xin Lian (*Andrographis paniculata* (Burm. F.) Nees)	HCT-116	–	Proliferation ↓, Migration ↓, Invasion ↓, Apoptosis ↑, Block cell cycle	↓: CDK2, CyclinB1, Cli1, SMO	Hedgehog	[251]
*Ganoderma atrum* polysaccharide (PSG-1)	Ling Zhi (*Ganoderma lucidum* (Leyss.ex Fr.) Karst.)	–	CT26 bearing mice	Tumor growth ↓, Immunity ↑	↑: NO, TNF-α, IL-1β, NF-κB, TLR4, p-p38 ↓: IκBα	TLR4-dependent P38 MAPK	[264]

**Table 2:** Effects of Chinese herbal formulas on preventing and treating CRC

Formula	Herbs	Experiments		Effects	Molecular mechanism	Signal pathways	Refs.
		In vitro model	In vivo model				
Gegen Qinlian Decoction (GQD)	Ge Gen (*Pueraria lobata* (Willd.) Ohwi), Gan Cao (*Glycyrrhiza uralensis* Fisch.), Huang Qin (*Scutellaria baicalensis* Georgi), Huang Lian (*Coptis chinensis* Franch.)	–	CT-26 spleen transplanted mice	Proliferation ↓, Migration ↓	↑: p-PI3K, p-AKT, p-FOXO1, ABTB1	PI3K/AKT/FOXO1	[55]
Tounong Powder extracts (TNSEs)	Huang Qi (*Astragalus membranaceus* (Fisch.)), Dang Gui (*Aaugellica sinensis*(Oliv) Diels.), Chuan Xiong (*Ligusticum chuanxiong* Hort.), Zao Jiao Ci ( *Gleditsia sinensis* Lam.), Chuan Shan Jia (*Manis pentadactyia* Linnaeus)	LoVo	–	Proliferation ↓, Apoptosis ↑, Block cell cycle	↑: cleaved caspase-3,cleaved caspase-9 ↓: PI3K,p-AKT, p-mTOR, p-p70s6k1	PI3K/Akt	[56]
Jiedu Sangen decoction (JSD)	Pu Gong Ying Gen (*Taraxacum mongolicum*Hand. –Mazz.), Lu Gen (*Phragmites communis* Trin.), Mao Gen (*Perotis indica* (L.) Kuntze)	HCT-8/5-FU	–	Glycolysis ↓, Apoptosis ↑	↑: caspase-3,caspase-9, caspase-6, caspase-7 ↓: HIF-1α, PI3K AKT/p-AKT, HKII, Glut1	PI3K/ Akt/HIF-1α	[57]
Wu-Mei-Wan (WMW)	Wu Mei (*Prunus mume* (Sieb.) Sieb.et Zucc.), Huang Lian (*Coptis chinensis* Franch.), Xi Xin (*Asarum heterotropoides* Fr.Schmidt var.mandshuricum(Maxim.)kitag.), Gui Zhi (*Cinnamomum cassia* Presl), Dang Shen (*Changium smyrnioides* Wolff ), Fu Zi (*Aconitum carmichaeli* Debx.), Hua Jiao (*Zanthoxylum schinifolium* Sieb.et Zucc.), Gan Jiang (*Zingiber officinale* Rosc.), Huang Bai (*Phellodendron chinense* Schneid.), Dang Gui (*Aaugellica sinensis*(Oliv) Diels.).	–	AOM/DSS induced CAC model mice	Proliferation ↓	↓: IL-6, p65, p-STAT3	NF-κB/IL-6/STAT3	[77]
Zuo-Jin-Wan (ZJW)	Huang Lian (*Coptis chinensis* Franch.), Wu Zhu Yu (*Evodia rutaecarpa* (Juss.) Benth.)	HCT-116/L-OHP	–	Drug-resistance ↓, Apoptosis ↑	↓: p-Akt (Ser473), p-NF-κB	PI3K/Akt/NF-κB	[78]
Yi-Qi-Fu-Sheng (YQFS)	Dang Shen (*Changium smyrnioides* Wolff ), Bai Zhu (*Atractylodes macrocephala* Koidz.), Fu Ling (*Poria cocos* (Schw.) Wolf), Gan Cao (*Gl. uralensis* Fisch.), Rou Dou Kou (*Myristica fragrans* Houtt), Ba Yue Zha (*Akebiaquinata*(Thunb.)Decne.)	HCT-116	–	Apoptosis ↑, Migration ↓, Invasion ↓	↓: p-ERK	ERK/MAPK	[101]
		–	HCT-116 xenograft mice	Tumor growth ↓	↓: ERK, MMP2, MMP9	ERK	
Qizhen capsule (QZC)	Huang Qi (*Astragalus membranaceus* (Fisch.)), Ren Shen (*Panax ginseng* C.A.Mey.), Da Qing Ye (*Isatis indigotica* Fort.), Chong Lou (*Paris polyphylla* Smith Var. yunnanensis (Franch.) Hand.-Mazz), Zhen Zhu (*Pteria martensii* (Dunker))	HCT-116	–	Apoptosis ↑	↑: cleaved caspase-9, cleaved caspase-3, Bax, NAG-1/GDF15, p-mTOR, p-MAPK/ERK、p-AMPK, p-p38 ↓: Bcl-2	MAPK/ERK	[102]
Geijigajakyak Decoction (GJD)	Rou Gui (*Cinnamomum cassia* Presl), Gan Cao (*Gl. uralensis* Fisch.), Shao Yao (*Raeonia lactiflora* pall.), Gan Jiang (*Zingiber officinale* Rosc.), Da Zao (*Ziziphus jujuba* Mill.)	HCT-116	–	Invasion ↓	↓: p-JNK, p-p38 MAPK	JNK p38 MAPK	[103]
Weichang’an (WCA)	Tai Zi Shen (*Peseudostellaria heterophylla* (Miq.)Pax ex pax et Hoffm.), Bai Zhu (*Atractylodes macrocephala* Koidz.), Fu Ling (*Poria cocos* (Schw.) Wolf), Ban Xia (*Pinellia ternata* (Thunb) Breit.), Chen Pi (*Citrus reticulata* Blanco), Qing Pi (*Citrus reticulata* Blanco), Xia Ku Cao (*Prunella vulgaris* L.), Da Xue Teng (*Sargentodoxa cuneata* (oliv.) Rehd.et wi1s.), Teng Li Gen (*Actinidia chinensis* Planch.), Ye Pu Tao Teng (*Ampelopsis brevipedunculata* (Maxim.)Trautv.), Huang Lian (*Coptis chinensis* Franch.), Mu Li (*Ostrea gigas* Thunberg), Bi Hu (*Gekko*), Bai Bian Dou (*Dolichos lablab* L.), Lv E Mei (*Prunus mume* (Sieb.) Sieb.et Zucc.), Ji Nei Jin (*Gallus gallus domesticus* Brisson)	HCT-116	–	Migration ↓, Invasion ↓,	↑: ARHGAP25 ↓: MMP7, MMP9, ZEB1, β-catenin	Wnt/β-catenin	[126]
Zuo Jin Wan (ZJW)	Huang Lian (*Coptis chinensis* Franch.), Wu Zhu Yu (*Evodia rutaecarpa* (Juss.) Benth.)	SW408	–	Apoptosis ↑, Migration ↓, Invasion ↓, Block cell cycle	↑: 5-HTR1D, Axin1 ↓: Dvl2, p-GSK3β, LEF1, TCF4, MMP2, MMP7, ICAM-1, CXCR4	5-HTR1D-Wnt/β-catenin	[127]
Pai-Nong-San (PNS)	Zhi Shi (*Citrus aurantium* L.), Shao Yao (*Paeonia lactiflora* Pall.), Jie Geng (*Platycodon grandiflorus* (Jacq.) A. DC. )	–	AOM/DSS induced CAC model mice	Inflammation ↓, Gut microbiota ↑	↑: GSK3β, p-β-catenin ↓: p-GSK3β, β-catenin, c-Myc	Wnt	[128]
JianPi JieDu Recipe (JPJD)	Huang Qi (*Astragalus membranaceus* (Fisch.)), Bai Zhu (*Atractylodes macrocephala* Koidz.), Ye Pu Tao Teng(*Ampelopsis brevipedunculata* (Maxim.)Trautv.), Ba Yue Zha (*Akebiaquinata*(Thunb.)Decne.), Shi Jian Chuan (*Salvia chinensia* Benth.), Wu Zhu Yu (*Evodia rutaecarpa* (Juss.) Benth.)	LoVo	Orthotopic transplantation tumor mice (LoVo)	EMT ↓, Migration ↓, Invasion ↓	↑: E-cadherin ↓: p-Smad2/3, Snali	TGF-β/Smad 2/3	[194]
Modified Shenlingbaizhu Decoction (MSD)	Ren Shen (*Panax ginseng* C. A. Mey), Fu Ling (*Poria cocos* (Schw.) Wolf), Liao Ge Wang (*Wikstroemia indica* (L.) C. A. Mey), Bai Zhu (*Atractylodes macrocephala* Koid*z.*), Cu Ye Rong (*Ficus hirta* Vahl), Huo Tan Mu (*Polygonum chinense* L.), Ci Qiu (*Kalopanax septemlobus* (Thunb.) Koidz.),ErZhu (*Curcuma phaeocaulis* Val.), Yi Yi Ren (*Coix lacryma-jobi L.var.ma-yuen* (Roman.) Stapf), Chun Pi (*Ailanthus altissima* (Mill.) Swingle)	–	Orthotopic transplantation tumor mice (SW480)	Proliferation ↓, Migration ↓, Invasion ↓	↓: TβRI, CD133, Vimentin, OCT-4 A, SOX2	TGF-β/Smad	[195]
Pien Tze Huang (PZH)	She Xiang (*Moschus berezovskii* Flerov), Niu Huang (*Bos taurus domesticus* Gmelin), San Qi (*Panax notoginseng* (Burk.) F. H. Chen), She Dan (*Python molurus bivittatus* Schlegel)	CT-26	Orthotopic transplantation tumor mice (CT-26)	EMT ↓, Migration ↓, Invasion ↓	↑: E-cadherin ↓: N-cadherin, TGF-β, p-Smad2/3, p-Smad 4	TGF-β/Smad	[196]
Qingjie Fuzheng granule (QFG)	Bai Hua She She Cao (*Oldenlandia diffusa* (willd.) Roxb), Ban Zhi Lian (*Scutellaria barbata* D. Don), Mai Ya (*Hordeum vulgare* L.), Huang Qi (*Astragalus membranaceus* (Fisch.))	–	HCT-116 xenograft mice	Autophagy ↑, EMT ↓	↑: E-cadherin, LC3-II, Beclin-1 ↓: N-cadherin,, Vimentin, TWIST1, p62, PI3K/PI3K, p-AKT/AKT, p-mTOR/mTOR	mTOR	[226]
Jianpi Jiedu Decoction (JPJD)	Huang Qi (*Astragalus membranaceus* (Fisch.)), San Qi (*Panax notoginseng* (Burk.) F. H. Chens), Bai Zhu (*Atractylodes macrocephala* Koidz.), Fu Ling (*Poria cocos* (Schw.) Wolf), Yi Yi Ren (*Coix lacryma-jobi L.var.ma-yuen* (Roman.) Stapf), Tu Fu Ling (*Smilax glabra* Roxb.), Bai Hua She She Cao (*Oldenlandia diffusa* (willd.) Roxb), Ban Zhi Lian (*Scutellaria barbata* D. Don), Chong Lou (*Paris polyphylla Smith Var. yunnanensis* (Franch.) Hand.-Mazz), Mi Hou Tao (*Actinidia chinensis* Planch.), Gan Cao (*Gl.* Fisch.)	HCT-116	HCT-116 xenograft mice	Migration ↓, Invasion ↓, Angiogenesis ↓, Tumor growth ↓	↓: mTOR, HIF-1α, VEGF, phospho-p70S6K, p-4E-BP1	mTOR/HIF-1α/VEGF	[227]
Qingjie Fuzheng Granules (QFG)	Bai Hua She She Cao (*Oldenlandia diffusa* (willd.) Roxb), Ban Zhi Lian (*Scutellaria barbata* D. Don), Mai Ya (*Hordeum vulgare* L.), Huang Qi (*Astragalus membranaceus* (Fisch.))	–	HCT-116 xenograft mice	Proliferation ↓, Apoptosis ↑, Angiogenesis ↓	↑: Bax/Bcl-2, p21 ↓: SHH, Smo, Cli1, Ki-67, CyclinD1, CDK4, VEGF-A, VEGFR-2	Sonic Hedgehog	[252]

In addition, signaling pathways such as Notch [272], is closely associated with CRC progression, and further in-depth studies are needed to prevent and treat CRC through these pathways. The existing reports have only discussed a single signaling pathway and related genes in the progression of CRC, without involving the interaction between signaling pathways. Most of the current studies have revealed the therapeutic effect of TCM on CRC. In vitro cellular models should be combined with in vivo animal models as much as possible, so that the two models can complement each other and jointly promote research on the pathogenesis of CRC and the prevention and treatment of TCM. TCM contains a wealth of resources that should be thoroughly investigated to determine whether other Chinese medical methods such as acupuncture, moxibustion, and acupoint injection have an intervention effect on CRC-related signaling pathways, providing more theoretical support for the use of TCM in malignant tumors. Dialectical analysis of the disease and a grasp of the holistic view of TCM and personalized medicine in the treatment of the disease are lacking. Future studies should pay further attention to the synergistic effect of multiple signaling pathways regulated by TCM on anti-CRC and increase support for the clinical transformation of TCM to provide new ideas and references for the application of TCM in the prevention and treatment of CRC.

## Data Availability

No data was used for the research described in the article.

## Abbreviations

CRC: Colorectal cancer
TCM: Traditional Chinese medicine
IBD: Inflammatory bowel disease
K-Ras: Kirsten rat sarcoma
MSI: Microsatellite-unstable/instability
CDX2: Caudal-related homeobox 2
MAPK: Mitogen-activated protein kinase
PI3K: Phosphatidylinositol 3-kinase
Akt: Protein kinase-B
RTKs: Receptor tyrosine kinases
EGFRs: Epidermal growth factor receptors
EMT: Epithelial–mesenchymal transition
WOG: Wogonin
LC3II: light chain 3-II
Bax: Bcl-2-associated X
Bcl-2: B-cell lymphoma-2
p-STAT3: Phosphorylated signal transducer and activator of transcription 3
EMD: Emodin
TP: Triptolide
PD: Platycodin D
GOD: Gegen Qinlian Decoction
p-FOXO1: Phosphorylated forkhead box transcription factor O1
ABTB1: Ankyrin repeat and BTB/POZ domain containing protein 1
TNSEs: Tounong powder extracts
p-mTOR: Phosphorylated mechanistic target of rapamycin
HIF-1α: Hypoxia-inducible factor-1α
GLUT1: Glucose transporter type 1
JSD: Jiedu Sangen decoction
NF-κB: Nuclear factor kappa-beta
IKK: IκB kinase
ERK: Extracellular signal-regulated kinase
ORI: Oridonin
AP-1: Activating protein-1
BE: Baicalein
BBR: Berberine
IL-1β: Interleukin-1β
TNF-α: Tumor necrosis factorα
MMP9: Matrix metallopeptidase 9
COX2: Cyclo-oxygenase-2
ICAM-1: Intercellular adhesion molecule 1
CXCR4: C-X-C motif chemokine receptor 4
VEGF: Vascular endothelial growth factor
WMW: Wu Mei Wan
CAC: AOM/DSS-induced colitis-associated CRC
IL-6: Interleukin 6
ZJW: Zuo Jin Wan
JNK: Jun N-terminal kinase
MAP2K: MAPK kinase
MAP3K: MAPK kinase-kinase
ATF2: Transcription factor 2
FRA: Fos-related antigen
Rh1: Ginsenoside Rh1
PT: Podophyllotoxin
ROS: Reactive oxygen species
YQFS: Yi-Qi-Fu-Sheng
QZC: Qizhen capsule
NAG-1: Nonsteroidal anti-inflammatory drug-activated gene-1
GDF15: Growth differentiation factor-15
AMPK: AMP-activated protein kinase
GJD: Geijigajakyak decoction
GSK3β: Glycogen synthase kinase 3beta
CK1: Casein kinase 1
APC: Adenomatous polyposis coli
TCF: T-cell transcription factor
LEF: Lymphoid enhancer factor
CTNNB1: β-catenin
TCF7L2: Transcription factor-7-like-2
GGC: Ginkgolide C
WCA: Weichang’an
ARHGAP25: Rho GTPase activating protein 25
ZEB1: Zinc finger E-box binding homeobox 1
5-HTR1D: 5-hydroxytryptamine receptor D
Dvl2: Dishevelled 2
LEF1: Lymphoid enhancer-binding factor 1
TCF4: Transcription factor 4
TK: Tyrosine kinase
AR: Am-phiregulin
EGF: Epidermal growth factor
TGF-α: Transforming growth factorα
GA: Gambogic acid
SOX2: SRY-box transcription factor 2
CD133: Cluster of differentiation 133
Bmi-1: B-lymphoma Mo-MLV insertion region 1
ZFP36: Zinc-finger protein 36
NCTD: Norcantharidin
UA: Ursolic acid
LZ: Ligustrazine
GLPs: *Ganoderma lucidum* polysaccharides
TGF-β: Transforming growth factor-β
TGFBR2: Type II TGF receptor
ZO-1: Zona occludens 1
OM: Oxymatrine
α-SMA: α-smooth muscle actin
PAI-1: Plasminogen activator inhibitor-1
CSCs: Colorectal cancer stem cells
mTORC1: mTOR complex1
mTORC2: mTOR complex2
QFG: Qingjie Fuzheng granule
JPJD: Jianpi Jiedu Decoction
p-p70S6K: Phosphorylation ribosomal protein S6 kinase
p-4E-BP1: Phosphorylation 4E binding protein 1
HH: Hedgehog
SHH: Sonic Hedgehog
DHH: Desert Hedgehog
Ptc: Patched
Smo: Smoothened
PTCH1: patched 1
TSN: Toosendanin
TME: Tumor microenvironment
IL-2: Interleukin-2
IFN-γ: Interferon-γ
IL-6: Interleukin-6
NK: Natural killer
TAMs: Tumor-associated macrophages
ICIs: Immune checkpoint inhibitors
PSG-1: *Ganoderma atrum* polysaccharide
NO: Nitric oxide
TLR4: Toll-like recepetor 4
IDO1: Indoleamine 2,3-dioxygenase 1
STAT1: Transcription 1
